# ATP-Charged Nanoclusters Enable Intracellular Protein Delivery and Activity Modulation for Cancer Theranostics

**DOI:** 10.1016/j.isci.2020.100872

**Published:** 2020-01-31

**Authors:** Zhanwei Zhou, Qingyan Zhang, Ruoxi Yang, Hui Wu, Minghua Zhang, Chenggen Qian, Xiangzhong Chen, Minjie Sun

**Affiliations:** 1 State Key Laboratory of Natural Medicines, Department of Pharmaceutics, China Pharmaceutical University, 24 Tong Jia Xiang, Nanjing 210009, P. R. China; 2 Multi-Scale Robotics Lab, Institute of Robotics & Intelligent Systems, ETH Zurich, Tannenstrasse 3, CLA H11.1, CH-8046 Zurich, Switzerland

**Keywords:** Medical Imaging, Catalysis, Cancer, Nanostructure

## Abstract

Protein drugs own a large share in the market and hold great prospects for the treatment of many diseases. However, the available protein drugs are limited to the extracellular target, owing to the inefficient transduction and activity modulation of proteins targeting intracellular environment. In this study, we constructed ATP-charged platforms to overcome the above-mentioned barriers for cancer theranostics. The phenylboronic acid-modified polycations (PCD) were synthesized to assemble with enzymes and shield its activity in the blood circulation. When the PCD nanoclusters reached tumor site, they effectively transported the enzymes into the cells, followed by recovering its catalytic activity after being charged with ATP. Importantly, the cascaded enzyme systems (GOx&HRPA) selectively induced starvation therapy as well as photoacoustic imaging of tumor. Our results revealed that the intelligent nanoclusters were broadly applicable for protein transduction and enzyme activity modulation, which could accelerate the clinical translation of protein drugs toward intracellular target.

## Highlights

• ATP-charged nanoclusters capable of enzyme activity modulation
• Universal platform for intracellular protein delivery
• Selective cellular cascaded catalysis for cancer theranostics
• Photoacoustic (PA) imaging and starvation therapy of breast cancer

## Graphical Abstract

**Figure:**
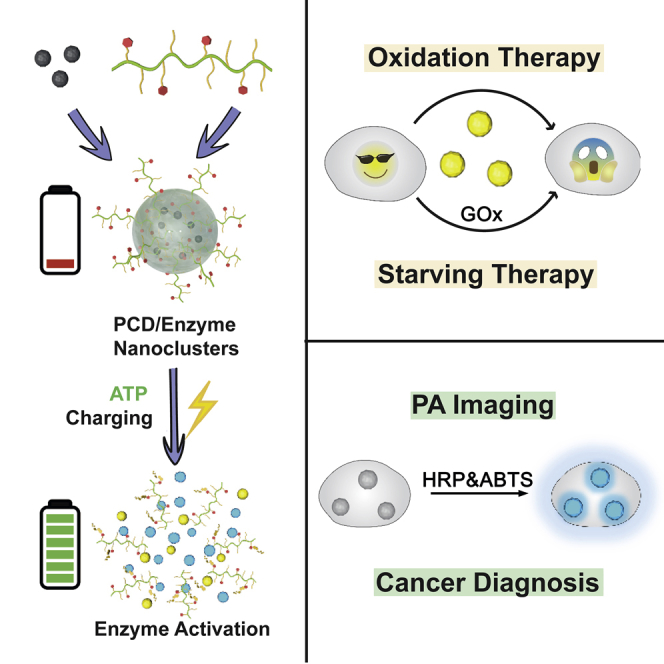

## Introduction

Proteins drugs have been widely developed for the diagnosis and treatment of various diseases (cancer, diabetes, or virus infection) owing to their high bioactivity and target selectivity (Kaspar and Reichert, 2013, Krejsa et al., 2006, Li et al., 2017b, Mo et al., 2014, Sangsuwan et al., 2019). However, most of the clinically approved protein drugs are developed toward extracellular targets (Pakulska et al., 2016, Zelikin et al., 2016). It is a pity that few proteins drugs toward intracellular environment have been developed because of the poor membrane-penetrating ability of naked proteins (Qian et al., 2018, Zhang et al., 2018, Zhu et al., 2018). Moreover, many proteins targeting intracellular environment like toxic enzymes (GOx, RNase A, DNase I, trypsin, etc.) often caused serious side effects for the high activity in blood circulation (Liu et al., 2018a, Zhao et al., 2017).

In recent decades, covalent modification or assembly with carriers has been applied to improve the intracellular delivery of proteins (Lee et al., 2009, Lu et al., 2014, Scaletti et al., 2018, Tang et al., 2013). Unfortunately, it was difficult to maintain the balance between the activity in blood circulation and intracellular environment, which means that the incompact packaging during delivery often leads to the leakage of these toxic enzymes and unspecific catalysis, whereas tight packaging could result in attenuated enzymatic activities due to the changing of secondary structure and specific surface area. Thus, how to transport these enzymes into the cell and achieve specifically intracellular catalysis is still challenging (Liu et al., 2018b).

**Scheme 1:**
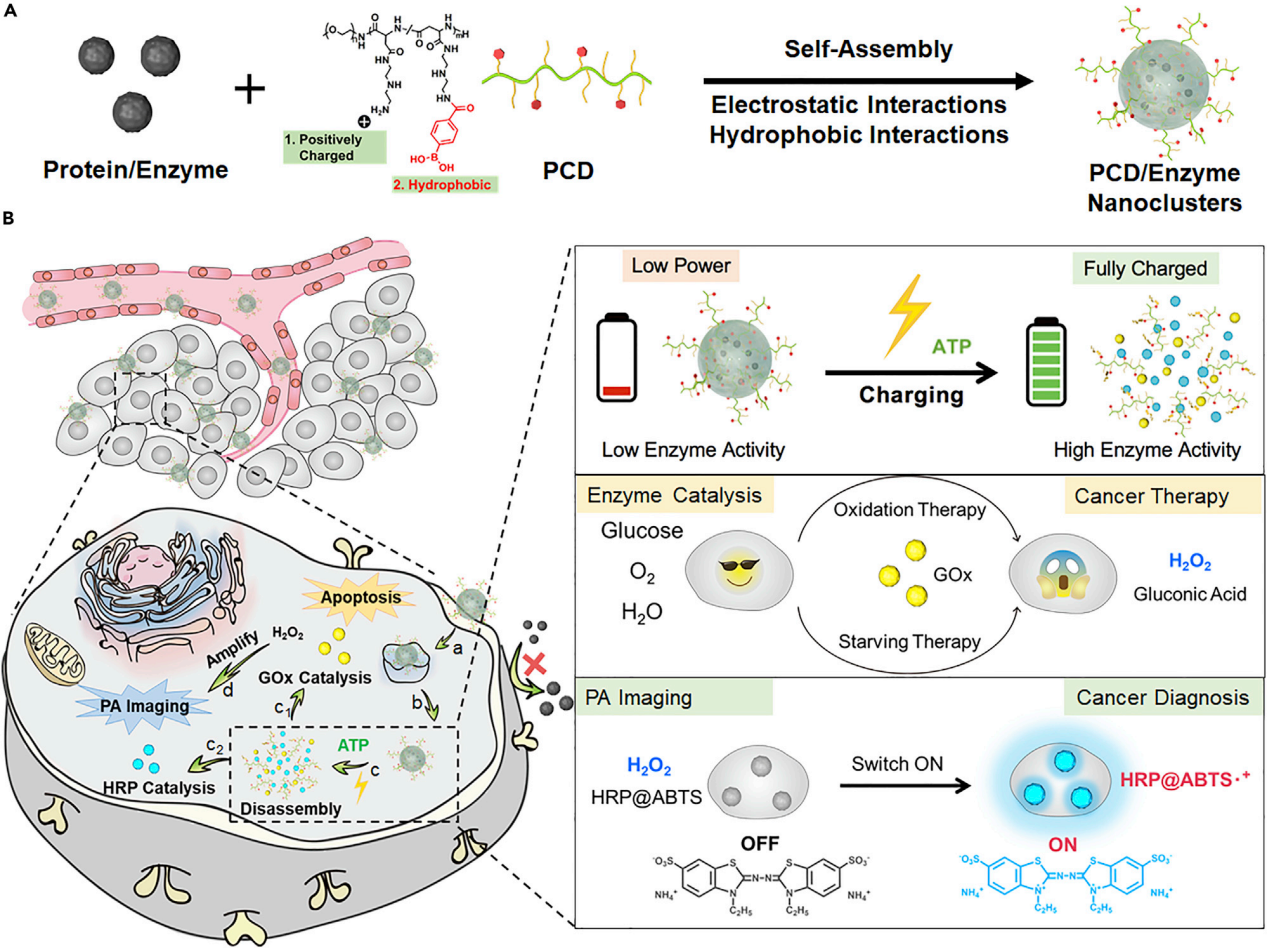
Schematic Illustration of ATP-Charged Nanoclusters with Cascade Catalysis for Amplifying the PA Imaging-Guided Cancer Therapy (A) The preparation procedure of PCD/enzymes nanoclusters. (B) *In vivo* behaviour of the nanocluster: (a) Cell uptake of the nanoclusters; (b) endosomal escape of the nanoclusters; (c) ATP-charged nanocluster activation, enzymes release, and activity recovery: (c_1_) GOx catalyzed the depletion of glucose and the generation of H_2_O_2_; (c_2_) HRP catalyzed the transformation of inactive ABTS to active ABTS·+ in the presence of H_2_O_2_; (d) the generation of H_2_O_2_ by GOx amplified the PA imaging.

Intelligent dynamic assemblies have attracted great attention because they can achieve temporal and spatial controlled activation of therapeutic payloads by predefined program (Lu et al., 2014). Here, we propose an intelligent vehicle, which has a simple structure but can still effectively realize site-specific activation of intracellularly targeted enzymes by shielding their activity in the blood circulation, efficiently transporting them to the cytosol, and recovering their activity selectively at the target site upon physiological trigger ATP (Scheme 1) (Biswas et al., 2013).

To achieve these goals, a polycationic polymer mPEG-b-poly(2-[(2-aminoethyl)amino]ethylaspatamide) (pDET) was synthesized, and modified with phenylboronic acid (PBA) (PCD, represents polymers), to assemble with enzymes such as GOx and ABTS (2, 2′-azino-bis(3-ethylbenzothiazoline-6-sulfonic acid))-loaded HRP (horseradish peroxidase) (named as HRPA) by electrostatic interaction, and to form enzyme nanoclusters. The assembled PCD/enzyme nanoclusters have a relative low enzyme activity (low-power state) to avoid unspecific catalysis in blood circulation. Besides, the formation of relatively large-sized nanoparticles could extend the blood circulation time and enhance the tumor accumulation of enzymes. When the nanoclusters reached tumor site, the assembly with carriers could promote the intracellular transduction of enzymes. Once in the cell, the diols on the pentose ring of ATP were able to form dynamic chemical bonds with PBA for accelerating the charge and hydrophobic property reversal of PCD, resulting in disassembly of the nanoclusters and release of the toxic enzymes (charging process, high-power state, Scheme 1c). As a result, the high-activity GOx could catalyze the depletion of glucose for tumor starvation therapy, and in the meantime, the production of H_2_O_2_, which acts as the substrate of HRPA to obtain active ABT·+ for cascaded amplifying photoacoustic (PA) imaging for diagnosis. The ATP-charged nanoclusters are believed to significantly improve the cytosolic transduction of proteins, especially for the enzyme activity modulation and tumor selective catalysis, and hence promote the diagnosis and therapeutic efficacy of cancers. Moreover, the Shield-Transport-Recover (defined as STR) intelligent cluster is a universal platform that can not only deliver the current enzymes but also be adapted to other enzyme systems.

## Result and Discussion

### Preparation and Characterization of Nanoclusters

**Figure 1:**
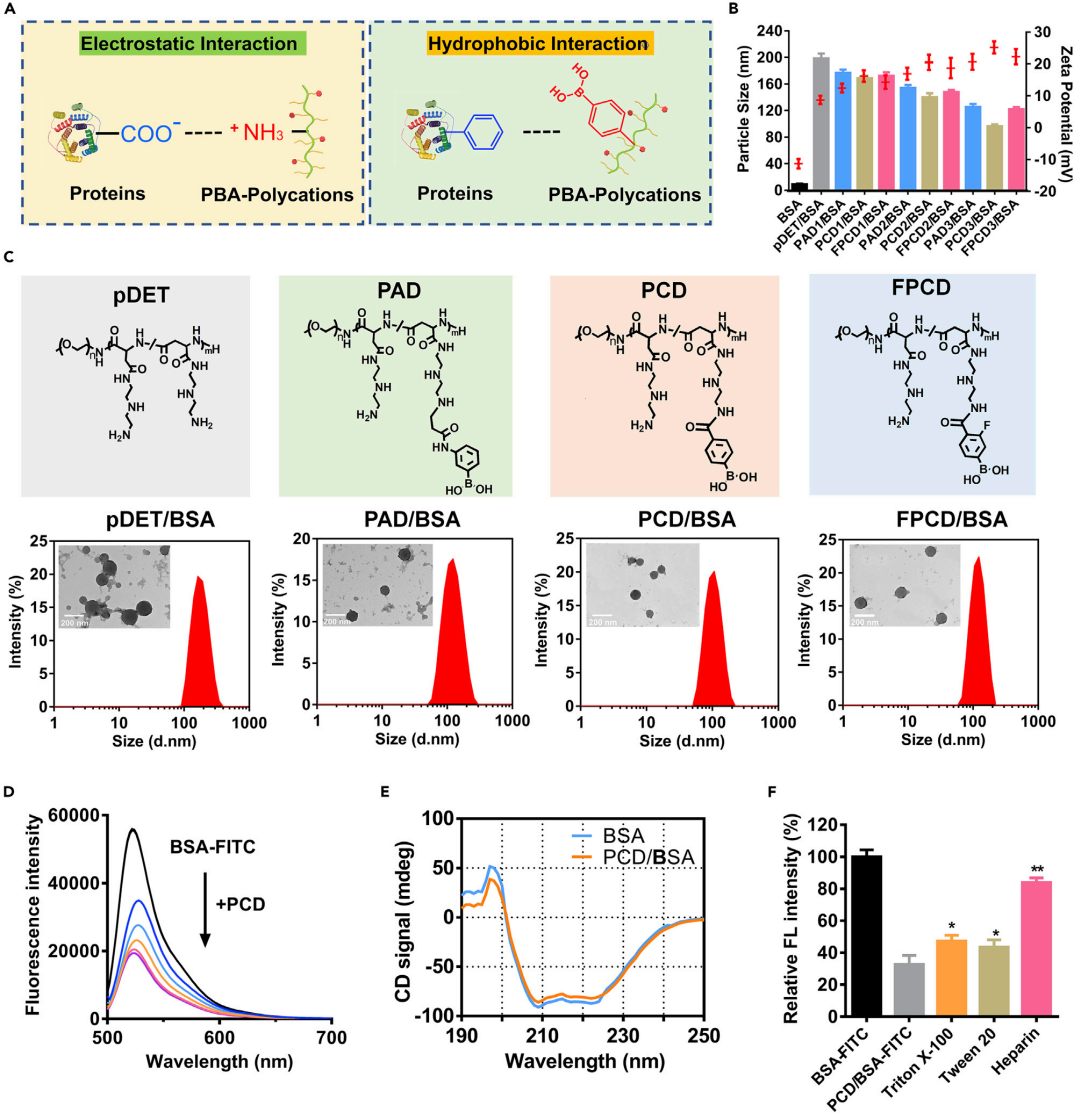
Characterization of the Protein Nanocluster (A) Mechanism illustration of the interactions between proteins and PBA-polycations. (B) Particle sizes and zeta potentials of naked BSA, pDET/BSA, PAD/BSA, PCD/BSA, and FPCD/BSA with series PBA modification ratios, where the number 1 means low DS polymer, 2 means moderate, and 3 means high DS polymer (n = 3, mean ± SD). (C) Chemical structures of the polymers, size distribution, and morphology observation of pDET/BSA, PAD/BSA, PCD/BSA, and FPCD/BSA nanoclusters (PAD3, PCD3, and FPCD3 were chosen as the representative polymers). (D) Fluorescence spectrum of BSA-FITC solution with increasing PCD added. (E) Circular dichroism spectrum of BSA and PCD/BSA. (F) Relative fluorescence intensity of BSA-FITC, PCD/BSA-FITC, and PCD/BSA-FITC treated with Triton X-100, Tween 20, or heparin (n = 3, mean ± SD). ∗p < 0.05, ∗∗p < 0.01. See also Table S1 and Figure S1–S7.

BSA was first applied to evaluate the formation of nanoclusters. As shown in Figure 1A, electrostatic and hydrophobic interactions may be involved in the binding between protein and PBA-modified polycations owing to the negatively charged and hydrophobic domain of proteins. As known to us, the pKa of PBA would affect the hydrophobicity and the binding ability with diol-containing molecules (Matsumoto et al., 2003, Matsumoto et al., 2012). Therefore, we would like to optimize the PBA functional polycations with different pKa as well as the different substitution degrees, where pDET was used as the model polycation. Polycations modified with three kinds of PBA were evaluated here (Figures S1–S6): (1) 3-(acrylamido) PBA-modified pDET was named as PAD with a pKa of 8.3; (2) 4-carboxyphenylboronic acid-modified pDET was named as PCD with a pKa of 7.8; and (3) 3-carboxy-4-fluorophenylboronic acid-modified pDET was named as FPCD with a pKa of 7.2.

As shown in Figure 1B, the particle size decreased with the higher PBA grafting ratio, whereas the zeta potential increased, indicating the role of PBA modification in compressing the particles and enhancing the charge density. Next, the size distribution and morphology observation of the representative nanoclusters were performed (pDET/BSA, PAD3/BSA, PCD3/BSA, and FPCD3/BSA). The results indicated that all the nanoclusters had spherical structure where pDET/BSA exhibited larger size and poor dispersion compared with the PBA-modified nanoclusters (Figure 1C). Here, the PCD/BSA was chosen as the representative nanocluster for further characterization.

For aggregation-caused quenching study (Chung et al., 2014), the BSA was labeled with fluorescein isothiocyanate (FITC) and the PCD was gradually added into the BSA-FITC solution; as obvious fluorescence quenching was observed (Figure 1D), reflecting the formation of BSA nanoclusters. Besides, the secondary structure of the proteins was also detected by circular dichroism spectrum. The formation of nanoclusters only slightly affected the secondary structure of BSA (Figure 1E). To further study the potential binding mechanism between PCD and proteins, several competitive reagents were used to treat the PCD/BSA nanoclusters by observing the fluorescence recovering (Chung et al., 2014). As shown in Figure 1F, obvious florescence recovery was observed after adding the hydrophobic competitive agents Triton X-100 or Tween 20, demonstrating the participation of the hydrophobic interactions in the formation of nanoclusters. Moreover, sodium heparin with strong negatively charged ions could significantly result in the recovery of fluorescence to about 80% of free BSA-FITC. The results demonstrated that both hydrophobic and ionic interactions were involved in the formation of nanoclusters.

In addition, the stability of the PCD/BSA clusters was also evaluated. As shown in Figure S7, the particle size of clusters could be maintained stable for 24 h in the presence of DW (deionized water), serum, medium, or pH 6.8 environment (tumor extracellular environment).

### ATP-Triggered Charge Reversal of Polycations and Release of BSA-FITC

**Figure 2:**
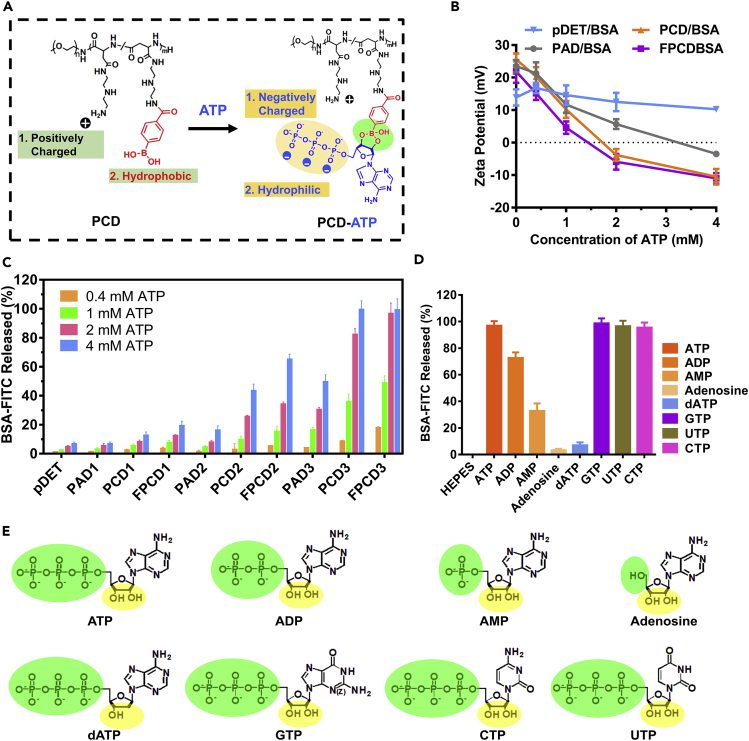
ATP-Dependent Protein Release from PCD/Protein Nanocluster (A) Schematic illustration of ATP-triggered charge reversal and hydrophobicity reversal of PCD. (B) BSA-FITC release from pDET/BSA-FITC, PAD/BSA-FITC, PCD/BSA-FITC, and FPCD/BSA-FITC treated with different concentrations of ATP (n = 3, mean ± SD). (C) Zeta potential detection of pDET/BSA, PAD/BSA, PCD/BSA, and FPCD/BSA nanoclusters with increasing ATP involvement (PAD3, PCD3, and FPCD3 were chosen as the representative polymers, n = 3, mean ± SD). (D) BSA-FITC release from PCD/BSA-FITC by incubation with different nucleotides (n = 3, mean ± SD). (E) Molecular formula of ATP, ADP, AMP, adenosine, dATP, GTP, CTP, and UTP. See also Table S1 and Figure S8.

The result of competitive experiment in Figure 1F inspired us to find a trigger that could simultaneously reverse the positive charge and the hydrophobicity of the carriers for efficient cargo release. Fortunately, adenosine triphosphate (ATP), the most abundant ribonucleotide in the cells, meets the above-mentioned requirements. The ATP molecules contain triphosphate group, which is strongly negatively charged and hydrophilic (Qian et al., 2016, Zhou et al., 2018a, Zhou et al., 2018b). In addition, intracellular ATP of tumor cells could reach as high as 1–10 mM, pretty higher than in the extracellular environment (0.1–0.4 mM) or blood circulation (<10 nM). The superior physicochemical properties and the biological gradient make ATP an ideal candidate for triggering nanocluster activation and protein release in the cytosol of tumor cells. Noteworthy, the intracellular ATP molecules were mainly used for providing energy for cell proliferation and metabolisms (Biswas et al., 2013). Therefore, herein, we made the hypothesis that ATP could be applied as the energy to charge the nanoclusters and therefore lead to the activation of nanoclusters. In detail, the chemical reaction between ATP and PBA could promote ATP binding and reverse the positive charge and hydrophobicity of PBA functional polycations, obtaining fully charged enzymes with high activity (Figure 2A).

First, the zeta potential change after treatment with ATP was evaluated (Figure 2B). The result confirmed that the PBA-unmodified polycations were insensitive to ATP, showing only slight zeta potential change after introduction of ATP. On the contrary, all the PBA-modified polycations exhibited improved ATP sensitiveness, where the zeta potential of PCD or FPCD dropped sharply and even recovered to negative when the concentration of ATP was higher than 1 mM. It revealed that PCD and FPCD showed better performance in response to ATP.

The ATP-charging process endowed the PCD-ATP polymer with zwitterionic properties, which was expected to result in protein repulsion and unpacking (Chen et al., 2005, Ladd et al., 2008, Shao and Jiang, 2015). The model protein BSA was labeled with FITC to make it convenient for release study (Lee et al., 2009, Zhou et al., 2019). Figure 2C indicated that increased BSA-FITC was released from the nanoclusters with increased PBA modification ratio. In addition, consistent with the zeta potential detecting data, PCD/BSA-FITC and FPCD/BSA-FITC were significantly more sensitive to ATP; nearly all proteins were released from the nanoclusters when the ATP concentration was higher than 2 mM, whereas PAD3 only released 50% proteins. It should also be mentioned that FPCD/BSA-FITC was excessively sensitive to ATP, leading to about 20% protein leakage in 0.4 mM ATP environment, mimicking the tumor extracellular environment. Thus, PCD3 was optimized as the best polymer and abbreviated as PCD for the whole study.

To further evaluate the importance of triphosphate in the ATP molecule, phosphate-free adenosine, monophosphate AMP, and diphosphate ADP were used as the negative control triggers. Expectantly, the decreasing number of phosphates led to less release of BSA-FITC (Figure 2D). Besides, the role of the chemical reaction between PBA in PCD and diols in ATP was evaluated by choosing diol-free trigger dATP (deoxyadenosine triphosphate). The result indicated that dATP could not trigger the BSA-FITC release, demonstrating the necessity of the diols in ATP. Furthermore, similar release was obtained after being treated with GTP, CTP, or UTP (the molecular formula is shown in Figure 2E) as with ATP, proving that the nucleotide base did not affect the release. Therefore, it could be concluded that the diols and the triphosphate are responsible for charging the nanoclusters and triggering protein release from the nanoclusters.

We also evaluated the release behavior of the GOx and HRP co-loaded PCD/GOx&HRPA nanoclusters. As shown in Figure S8, after incubation with glucose and ATP (mimic the intracellular environment), both the GOx-FITC and HRP-FITC could be effectively released from the nanoclusters with increasing ATP involvement. It also reflected that the generation of HRP@ABTS·+ had no influence on this process.

### ATP Charged the Nanoclusters and Modulated the Enzyme Activity

The assembly of enzymes into nanoclusters could significantly shield its catalytic activity, owing to the reduced specific surface area and the reduced chance to interact with substrate (Li et al., 2017a). The design of selective protein release from the ATP-charged nanoclusters was expected to modulate the enzyme activity to achieve selective catalysis in response to the abundant ATP environment. Here, three model enzymes (RNase A, GOx, and HRP) with different molecular weights (MWs) and isoelectric point (PIs) (Table 1) were applied and the enzyme activities of nanoclusters in the absence or presence of ATP were analyzed.

**Table 1:** Physicochemical Properties of the Model Enzymes

Enzyme	PI (Isoelectric Point)	MW (Molecular Weight)	Substrate	Product
RNase A (ribonuclease A)	9.6	13.7 kDa	RNA	Ribonucleotide
GOx (glucose oxidase)	4.2	160 kDa	Glucose, O_2_	Gluconic acid, H_2_O_2_
HRP (horseradish peroxidase)	7.2	40 kDa	ABTS	ABTS·+
			Amplex Red	Resorufin
β-Gal (β-galactosidase)	5.0	430 kDa	X-Gal	Dark blur product

**Figure 3:**
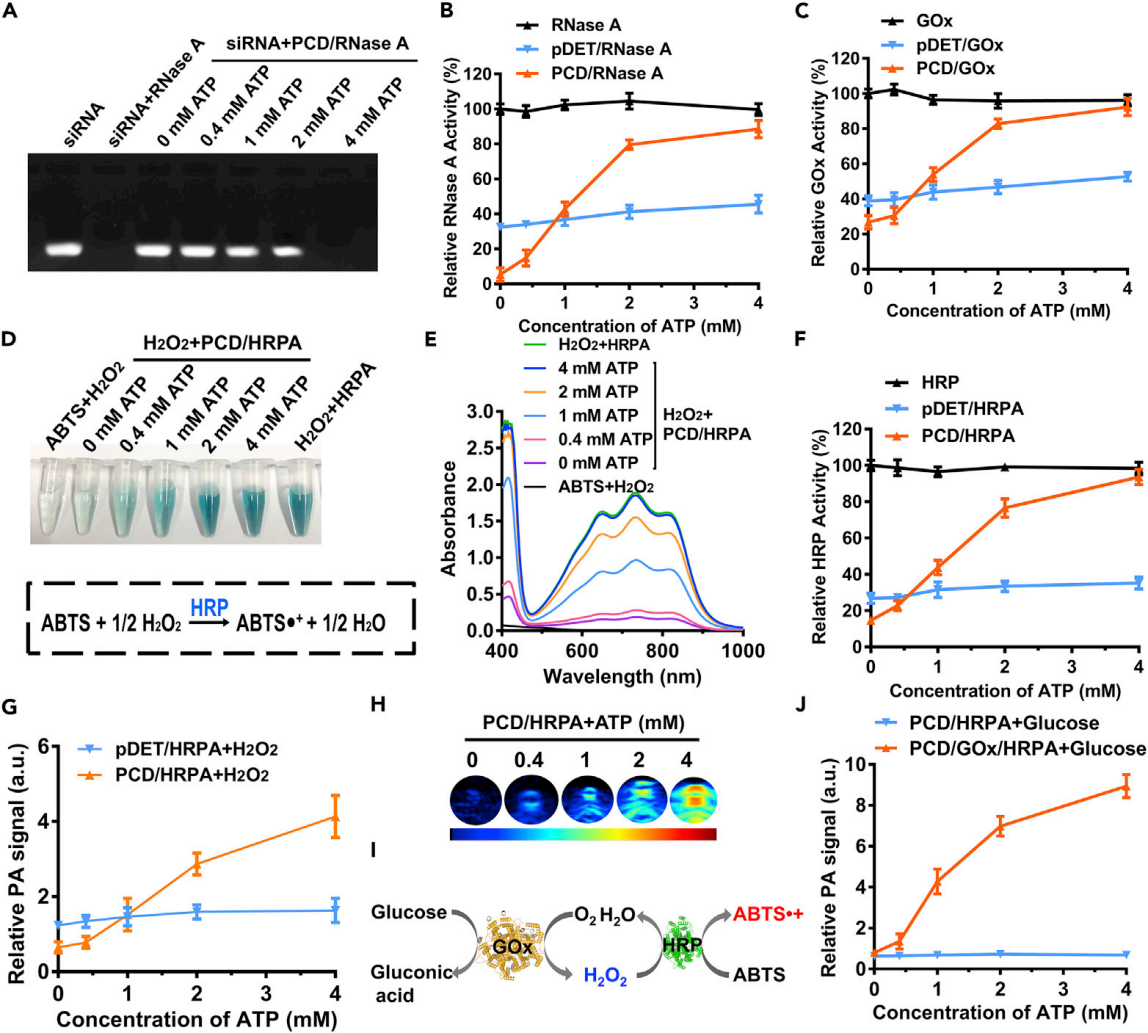
ATP-Dependent Enzyme Activity Modulation of PCD/Protein Nanocluster (A) Agarose gel electrophoresis analysis of siRNA incubated with free RNase A or PCD/RNase A with various ATP concentrations (0–4 mM). (B) Quantitative analysis of the relative RNase A activity of free RNase A, pDET/RNase A, and PCD/RNase A after incubation with ATP (n = 3, mean ± SD). (C) Relative GOx activity measurement of free GOx, pDET/GOx, and PCD/GOx on treatment with different concentrations of ATP (n = 3, mean ± SD). (D) Color changing of the ABTS + H_2_O_2_+PCD/HRPA in the presence of ATP and the equation of ABTS activation. (E) UV-vis spectrum of the samples shown in Figure 3D. (F) Relative HRP activity measurement of free HRP, pDET/HRP, and PCD/HRP on treatment with different concentrations of ATP (n = 3, mean ± SD). (G) Relative PA signal changing of pDET/HRPA and PCD/HRPA in the presence ATP (n = 3, mean ± SD). (H) PA images of the PCD/HRPA solution after incubation with series concentrations of ATP. (I) Reaction equation of cascaded amplifying catalysis between GOx and HRP. (J) Relative PA signal changing of PCD/HRPA or PCD/GOx/HRPA in the presence glucose and various ATP concentrations (n = 3, mean ± SD). See also Table S1.

As depicted in the agarose gel electrophoresis in Figure 3A, the free RNase A degraded all the small interfering RNA (siRNA) within 30 min, whereas the RNase A-loaded nanoclusters were inefficient in siRNA degradation owing to the shielding effect of nanoclusters. Interestingly, the catalytic activity recovered again after treatment with ATP, which was concentration dependent. The ATP-activated catalysis was also confirmed by quantitative analysis (Figure 3B). The relative RNase A activity of the nanoclusters was calculated by comparing with the free RNase A (100%) where the relative activity of PCD/RNase A recovered to as high as 90% in the presence of 4 mM ATP. Besides, it should also be noted that the activity of ATP-insensitive pDET nanoclusters was not recovered owing to the inefficient RNase A release.

The activity of GOx was evaluated by detecting the generation of H_2_O_2_. The GOx could catalyze the conversion of H_2_O, O_2_, and glucose into H_2_O_2_ and glucuronic acid (Fu et al., 2018). Expectantly, the dormant nanoclusters and the ATP-activated activity recovering of GOx were also validated as shown in Figure 3C, where the PCD/GOx displayed ATP-dependent H_2_O_2_ generation in the presence of glucose. The generated H_2_O_2_ held the potential to kill tumor cells by inducing oxidation stress.

We also studied the enzyme activity modulation on HRP. Here, the HRP could catalyze the photosilent ABTS into photoactive ABTS·+ in the presence of H_2_O_2_ (Figure 3D), inducing strong near-infrared (NIR) absorption (Abs_max_: 730 nm) (Yang et al., 2018). The ABTS was encapsulated in the HRP to form HRPA. Briefly, the H_2_O_2_ was treated with PCD/HRPA in the presence of various amounts of ATP, where the free HRPA was set as positive control and DW as the negative control. As shown in Figure 3D, the mixture solution turned deep green with increasing amount of ATP. The UV-vis absorbance spectrum of the samples in the range 400–1,000 nm was also measured, showing ATP-dependent NIR absorption enhancement (Figure 3E). The relative HRP activity was calculated by comparing the absorbance of free HRPA-treated sample at 730 nm, and the ATP-triggered enzyme activity recovery was observed from the result in Figure 3F.

Thus, the above-mentioned data demonstrated that the ATP-charged nanoclusters capable of activity modulation were generally applicable for most proteins with different MWs or PIs.

### ATP-Charged Cascaded Catalysis for Amplifying PA Imaging

The ATP-activated generation of strong NIR absorption endowed PCD/HRPA with the potential to be applied for PA imaging. The PA imaging signal of pDET/HRPA and PCD/HRPA in the presence of H_2_O_2_ and series concentrations of ATP was measured by LAZR PA Imaging System. In accordance with the UV-vis absorbance result, the PA signal of PCD/HRPA increased gradually with increasing amount of ATP added, whereas the PA signal of non-responsive pDET/HRPA did not change with the increasing ATP (Figure 3G). Besides, the ATP-activated PA images at 0.4–1 mM are also shown in Figure 3H. Notably, we found that H_2_O_2_ could act as the bridge between GOx and HRP because it was not only the product of GOx catalysis but also the substrate of HRP. Therefore, it inspired us to construct a cascaded enzyme system (PCD/GOx/HRPA) for amplifying the PA imaging signal (Figure 3I), where the GOx could continuously supply the H_2_O_2_ for promoting the activation of ABTS. The result shown in Figure 3J indicated that the PCD/GOx/HRPA nanoclusters could effectively catalyze the generation of PA signal in the presence of glucose by ATP-dependent enhancement, demonstrating the successful fabrication of the cascaded enzyme system. Moreover, we are excited to find that the PA signal of PCD/GOx/HRPA was much stronger than that of the PCD/HRPA + H_2_O_2_ solution, reflecting the cascade catalysis-induced remarkable amplification of the PA signal. As the control, the GOx-unloaded PCD/HRPA system could not generate PA signal, proving the importance of the GOx in the cascaded system. Hence, it demonstrated that the tumoral ATP could effectively charge the nanoclusters and activate the GOx and HRP; then the activated GOx catalyzed the generation of H_2_O_2_, which cascade amplified the activation of ABTS for PA imaging with the catalysis of HRP.

### Cell Uptake of the Nanoclusters

**Figure 4:**
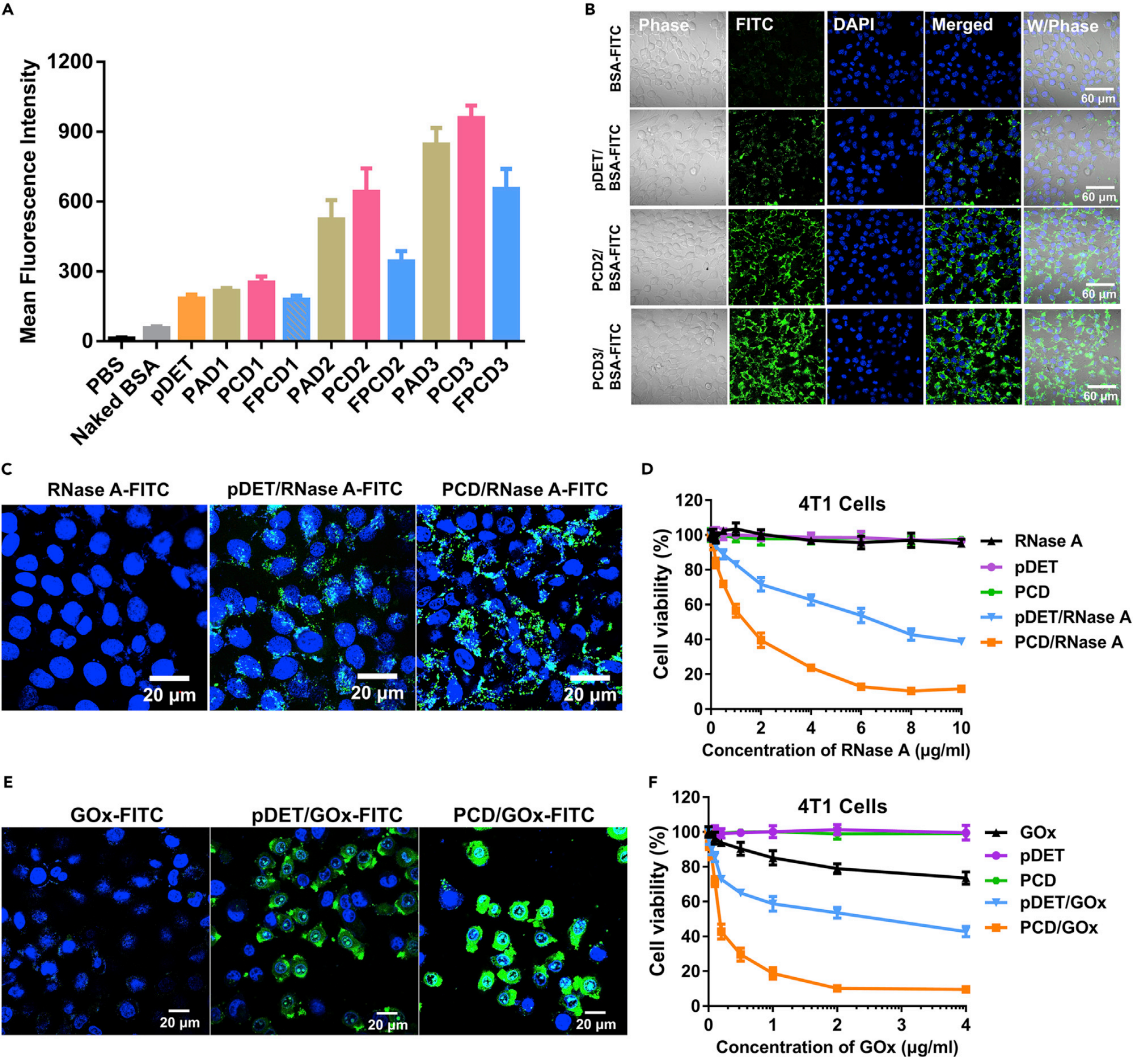
Cell Uptake and Tumor Cell Growth Inhibition of the PCD/Protein Nanocluster (A) Mean fluorescence intensity of 4T1 cells by FCM after incubation with BSA-FITC, pDET/BSA-FITC, PAD/BSA-FITC, PCD/BSA-FITC, and FCD/BSA-FITC of different PBA modification ratio (n = 3, mean ± SD). (B) CLSM observation of 4T1 cells after incubation with BSA-FITC, pDET/BSA-FITC, PCD2/BSA-FITC, and PCD3/BSA-FITC. (C) CLSM observation of 4T1 cells after incubation with RNase A-FITC, pDET/RNase A-FITC, and PCD/RNase A-FITC. (D) Cell viability of 4T1 cells incubated with pDET, PCD, RNase A, pDET/RNase A, and PCD/RNase A (n = 3, mean ± SD). (E) CLSM observation of 4T1 cells after incubation with GOx-FITC, pDET/GOx-FITC, and PCD/GOx-FITC. (F) Cell viability of 4T1 cells incubated with pDET, PCD, GOx, pDET/GOx, and PCD/GOx (n = 3, mean ± SD). See also Table S1 and Figures S9–S11.

Intracellular protein transduction was still a dilemma for successful application of protein drugs, especially for site-specific intracellular catalysis, because it was very difficult for the naked proteins to overcome the membrane barriers. The assembly with materials could efficiently assist to promote the cell uptake of proteins (Liu et al., 2018b). We first took the BSA-FITC as the model protein to optimize the series of polycations by flow cytometry (FCM) (Figure 4A). The free BSA-FITC exhibited pretty weak uptake in the 4T1 cells, and the PBA-unmodified pDET only slightly improved the cell uptake of proteins. Interestingly, it was obvious that the mean fluorescence intensity (MFI) increased sharply with increasing PBA modification ratio, proving the benefit of PBA modification on improving the cell uptake of proteins. Furthermore, it should be mentioned that the PCD nanoclusters showed the best performance on improving the cell uptake compared with PAD or FPCD. The relatively weaker uptake of PAD/BSA-FITC could be owing to the attenuated protein binding compared with PCD because the 3-amino phenylboronic acid tended to form intramolecular coordination bond between B and N. In addition, the FPCD had much stronger diol-binding ability compared with PCD, making it more easy to bind with glucose and hence hurdle the binding with sialic acid (SA), resulting in the diminished PBA-SA-mediated endocytosis of nanoclusters. In general, the FCM uptake result reflected that PCD3 was the best choice for further study. We also evaluated the intracellular distribution of the nanoclusters by confocal laser scanning microscopy (CLSM). As shown in the data of Figure 4B, there was nearly no green fluorescence signal in the cells after treatment with BSA-FITC, indicating the poor uptake of the free proteins. On the contrary, pretty strong green fluorescence signal was observed in the plasma after treatment with PCD3/BSA-FITC. Therefore, we could conclude that the formation of PCD nanoclusters was an effective strategy for improving the cytosolic transduction of proteins.

Apart from 4T1 cell lines, the cell uptake of PCD/BSA-FITC nanoclusters was also evaluated on L02 cells (human liver cells) and MCF-7 cells (human breast cancer cells). As displayed in Figure S9, the fluorescence in MCF-7 cells was much brighter than in L02 cells, which could be owing to the tumor cell-targeting ability of PBA-modified nanoclusters as previously described.

Subsequently, to further evaluate whether the PCD nanoclusters were broadly applicable for most of the proteins and could achieve enhanced tumor cell killing we took two representative bioactive proteins of RNase A and GOx for uptake and cell growth inhibition study. The uptake of PCD/RNase A-FITC shown in Figure 4C was much stronger than that of free RNase A-FITC or pDET/RNase A-FITC, indicating that the PCD nanoclusters were also suitable for the delivery of RNase A. In accordance with the uptake result, the free RNase A did not lead to cell death, whereas the PCD/RNase A nanoclusters displayed high toxicity on 4T1 cells, the IC50 of which was 1.2 μg/mL (Figure 4D). However, the IC50 of PBA-unmodified pDET was as high as 6.3 μg/mL, five times higher than that of PCD nanoclusters, further confirming the superiority of PCD on enzyme delivery and intracellular catalysis. The toxicity of the RNase A-loaded nanoclusters was also validated on normal cells (L02 cells). As shown in Figure S10, the toxicities of both pDET/RNase A and PCD/RNase A on L02 cells were much lower than those on 4T1 cells (ICD 50 > 10 μg/mL), which could be owing to the weaker cell uptake and slower enzyme release compared with tumor cells. It reflects the tumor-specific catalysis of the protein clusters.

In addition, the cytotoxic GOx was also assembled into the PCD nanoclusters for uptake and tumor cell killing study. GOx could selectively catalyze the conversion of glucose to cell toxic H_2_O_2_ and glucuronic acid for starvation and oxidation therapy. The improved cell transduction of GOx by PCD was validated in Figure 4E and the cytotoxicity-enhancing effect on 4T1 cell lines in Figure 4F. Different form RNase A, the free GOx could also generate H_2_O_2_ in the medium environment even if it was not internalized by the cells, leading to the slight cell toxicity. Excitingly, the assembly of PCD nanoclusters dramatically amplifies the cell killing effect of GOx on 4T1 cell lines. The IC50 of PCD/GOx was pretty low, about 0.15 μg/mL. Furthermore, the tumor-specific catalysis of GOx-loaded nanoclusters was also validated by detecting the toxicity on L02 cells (Figure S11). Similar to the RNase A result, the toxicity of GOx-loaded nanoclusters on L02 cells (ICD 50 > 4 μg/mL, 20- to 30-fold higher than 4T1 cells) was much lower than on 4T1 cells, which further demonstrated the cancer-specific catalysis of the clusters.

To conclude, these results demonstrated that the PCD nanoclusters could significantly promote the cell uptake of proteins and amplify the cell toxicity of the cytotoxic enzymes.

### Intracellular ATP-Dependent Enzyme Release and Activation

The high cytotoxicity of RNase A and GOx on 4T1 cells reflected that the loaded enzymes could effectively be released from the nanoclusters and thus activated for highly efficient catalysis. In addition, the ATP-responsive release of the protein from the nanoclusters had already been demonstrated in the solution sample. Thus, herein, we were desired to further evaluate whether the intracellular enzyme release and activation was intracellular ATP dependent.

**Figure 5:**
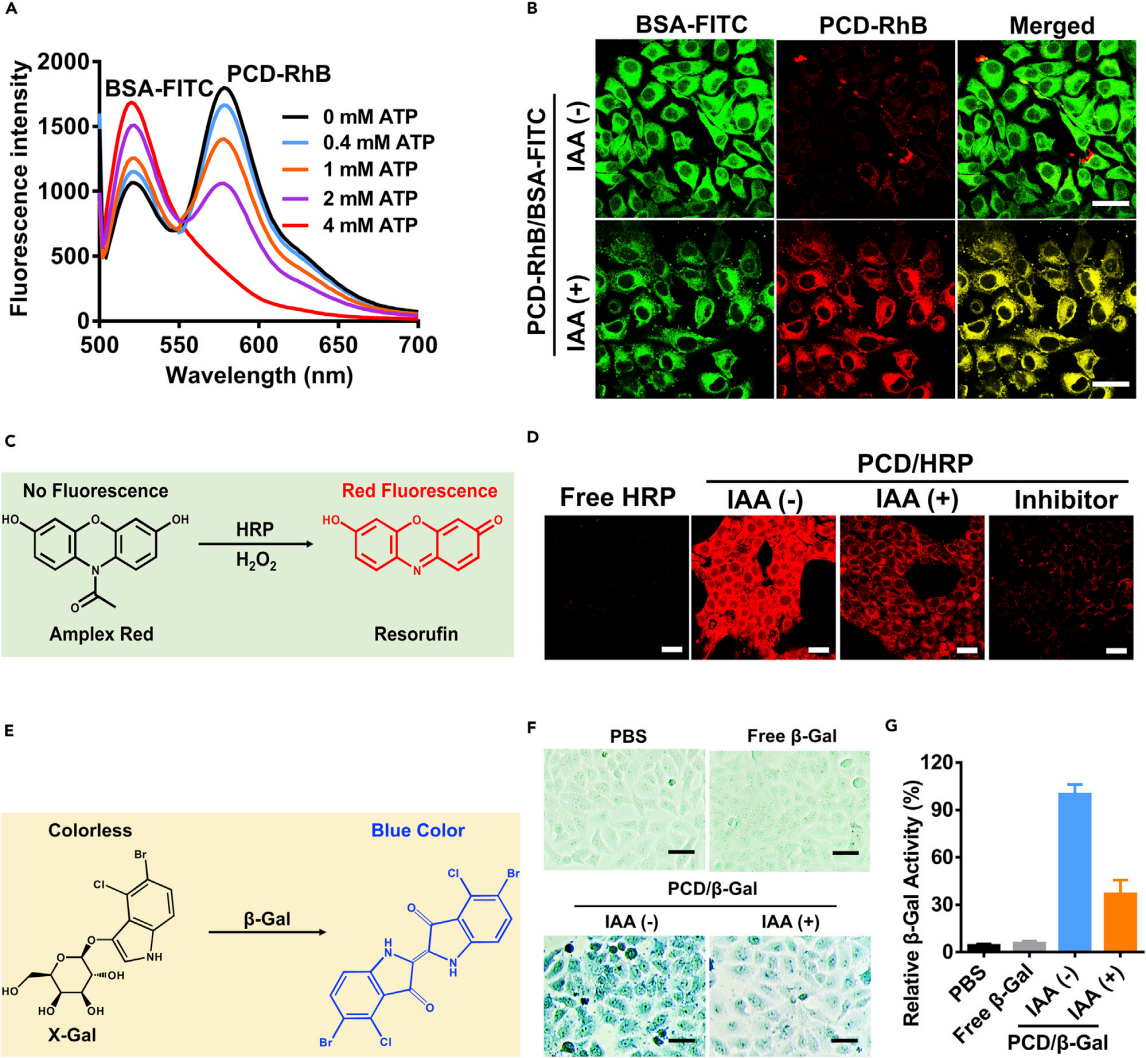
Intracellular ATP-Dependent Protein Release and Enzyme Activity Modulation of PCD/Protein Nanocluster (A) FRET spectrum of PCD-RhB/BSA-FITC in the presence of different concentrations of ATP (0 mM, 0.4 mM, 1 mM, 2 mM, and 4 mM). (B) Intracellular ATP-dependent protein release by FRET technology, where the FITC acted as the donor and the RhB acted as the acceptor (IAA: ATP generation inhibitor). Scale bar, 20 μm. (C) HRP could catalyze the conversion of nonfluorescent Amplex Red into resorufin with red fluorescence. (D) CLSM images of 4T1 cells transfected with free HRP, PCD/HRP with or without IAA, and HRP inhibitor NaN_3_, followed by being processed with H_2_O_2_ and Amplex Red. (E) β-Gal could catalyze the conversion of colourless X-Gal into blue product. (F) 4T1 cell images after treatment with PBS, free β-Gal, and PCD/β-Gal with or without IAA. Scale bar, 20 μm. (G) Quantitative analysis of relative β-Gal activity (n = 3, mean ± SD). See also Table S1 and Figures S12–S15.

The intracellular disassembly of nanoclusters and the release of enzymes were assessed by fluorescence resonance energy transfer (FRET) technology. The BSA was labeled with FITC as the donor, and the PCD was labeled with RhB as the acceptor. First, the generation of FRET signal was checked by scanning the fluorescence spectrum of PCD-RhB/BSA-FITC. As shown in Figure 5A, there are two obvious emission peaks (FITC: 520 nm, RhB: 580 nm) generated with the exciting laser of 480 nm. Interestingly, after the introduction of ATP, the peak at 580 nm underwent an attenuation process, indicating the release of BSA-FITC from the nanoclusters. Next, the intracellular ATP-dependent protein release was further verified (Figure 5B). Iodoacetic acid (IAA) was used to treat the cells for the construction of ATP deficiency model by inhibiting the glycolysis process and thus inhibiting the synthesis of ATP (Zhou et al., 2018a). The CLSM images displayed that the PCD-RhB signal was weak when the cells were not treated with IAA, indicating the disappearance of FRET signal and the release of BSA-FITC. On the contrary, the FRET signal remained strong (bright red fluorescence) after the cells were treated with IAA. It revealed that the release of proteins from PCD nanoclusters was mainly attributed to the intracellular abundant ATP.

In addition, the release behavior of PCD/BSA-FITC nanoclusters was also evaluated on normal cells (L02 cells). Without IAA treatment, there was still clear red fluorescence signal of the clusters, indicating the inefficient release of loaded enzymes in normal cells (Figure S12). These results demonstrated that the enzymes were quickly released in tumor cells but not in normal cells, reflecting the selectivity for tumor.

Keeping this in mind, we wanted to further check whether the ATP-induced protein release could promote the enzyme activation in the cells. The bioactive enzymes of HRP and β-Gal were applied to assess the intracellular enzyme activity modulation. HRP was capable of catalyzing conversion of the no fluorescence Amplex Red to fluorescent Resorufin in the presence of H_2_O_2_ (Figure 5C) (Dębski et al., 2016). The relative enzyme activity could be evaluated by the intensity of the red fluorescence signal. As displayed in the result, the free HRP was inefficient in catalyzing the emission of the dyes because of the poor cell uptake (Figure 5D), whereas the cells treated with PCD/HRP showed bright red fluorescence signal, reflecting the strong HRP catalysis activity in the cells. Meanwhile, after adding NaN_3_ (HRP inhibitor), the red fluorescence intensity dramatically decreased, validating that the generation of red fluorescence was attributed to the HRP catalysis. Moreover, the fluorescence intensity of IAA-treated cells also weakened, demonstrating the idea of ATP-dependent enzyme activation. Furthermore, to further validate the role of ATP-triggered enzyme release in activity recovery, ATP-insensitive pDET/enzyme clusters and ATP-sensitive PCD/enzyme clusters were both incubated in 4T1 cells with different enzyme concentrations to obtain the same uptake level as analyzed by flow cytometry (Figure S13A). As shown in Figure S13B, under the same uptake level, the enzyme activity of PCD/HRP cluster was much higher than that of pDET/HRP cluster, further validating that the higher activity of PCD/HRP group was attributed to the release.

In addition to 4T1 cells, the intracellular catalysis evaluation was also performed on L02 cells and MCF-7 cells. In accordance with the uptake and release result validated above, the L02 cells exhibited low intracellular HRP activity after transfection with PCD/HRP (Figure S14). On the contrary, the human breast cancer cells MCF-7 displayed strong red fluorescence after transfection with PCD/HRP, further confirming the specific catalysis of PCD nanoclusters in tumor cells.

The ATP-dependent enzyme activation was also evaluated on β-Gal. Specifically, the colorless substrate X-Gal could be catalyzed into blue products by β-Gal (Figure 5E), enabling it be applied for evaluating the intracellular β-Gal activity (Zhang et al., 2018). The 4T1 cells were treated with free β-Gal and PCD/β-Gal to transport the enzymes, and the deficient ATP model was constructed by incubation with IAA. After treatments, the cells were stained by *in situ* β-Gal staining kit according to the operating manual where the intensity of the blue color represented the intracellular β-Gal activity. The PCD/β-Gal-treated cells showed much deeper blue staining than the free β-Gal-treated cells, whereas the color turned dimmer after IAA incubation (Figure 5F). Thus, it also validated the ATP-dependent activation of β-Gal and the highly efficient intracellular catalysis of PCD nanoclusters. The relative β-Gal activity was also quantitatively analyzed by ImageJ analysis of the staining images where the relative β-Gal activity of the IAA-treated cells was less than 40% versus that of the IAA-untreated cells (Figure 5G).

Stability of the nanoclusters was very important for successful enzyme delivery, including in the presence of serum, under different temperature or in the tumor acidic microenvironment. Here, PCD/GOx was chosen as model cluster for evaluation, where the GOx activity was indicated by detecting the intracellular H_2_O_2_ generation via 2'-7'dichlorofluorescin diacetate (DCFH-DA) staining. As shown in Figure S15, the increasing FBS content from 0% to 30% only exhibited weak influence on enzyme activity, displaying good serum stability of PCD/GOx nanoclusters. More importantly, the acidic environment of tumor (pH 6.5 or 5.5) could promote intracellular catalysis compared with physiological environment (pH 7.4). Besides, temperature also has influence on intracellular catalysis. The enzyme activity under 25°C was much lower than that under 37°C or 43°C, indicating that physiological temperature (37°C) or relatively higher temperature (43°C) was beneficial for intracellular catalysis of PCD/GOx. To conclude, the above-mentioned data demonstrated that the PCD/GOx clusters could effectively achieve intracellular catalysis under tumor environment.

### *In Vivo* Biodistribution, Tumor Targeting, and Blood Circulation

**Figure 6:**
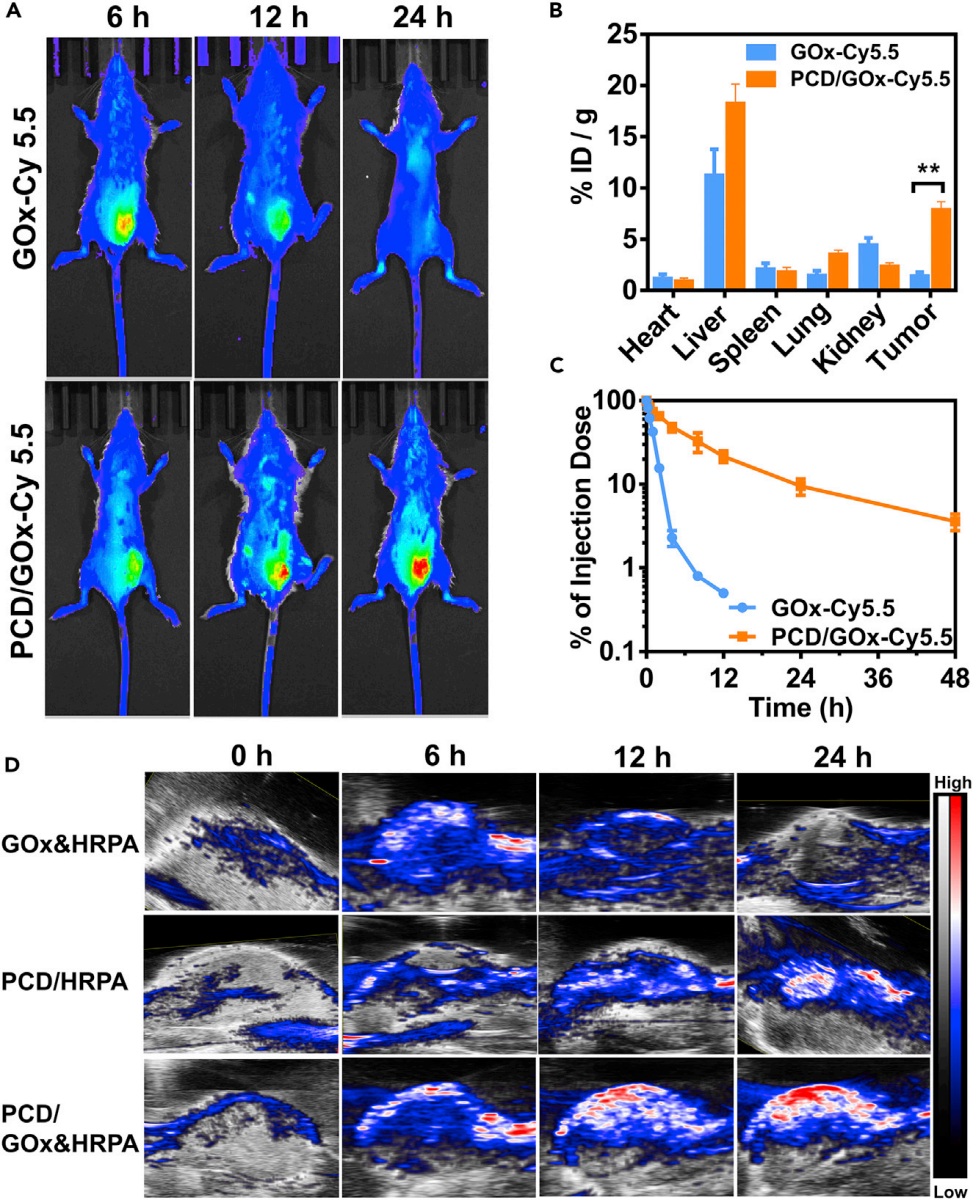
Biodistribution and PA Imaging of PCD/Protein Nanoclusters in 4T1-Bearing Mice (A) *In vivo* fluorescence images of 4T1-bearing mice after intravenous injection with GOx-Cy5.5 or PCD/GOx-Cy5.5. (B) Biodistribution of GOx-Cy5.5 and PCD/GOx-Cy5.5 at 24 h post-injection by detecting the Cy5.5 fluorescence intensity in the tissue lysates (n = 3, mean ± SD). ∗∗p < 0.01. (C) Blood circulation monitoring of GOx-Cy5.5 and PCD/GOx-Cy5.5 (n = 3, mean ± SD). (D) *In vivo* PA imaging of the 4T1-bearing mice after treatment with GOx&HRPA, PCD/HRPA, or PCD/GOx&HRPA. See also Table S1.

The *in vivo* biodistribution and tumor targeting of the PCD/GOx nanoclusters were evaluated by labeling GOx with NIR fluorescence probe Cy5.5 to make it visible under *in vivo* imaging system. As depicted in Figure 6A, free GOx-Cy5.5 showed the strongest tumor accumulation at 6 h post-injection and the fluorescence became dim with extended circulation time, which could be owing to the ultra-small size of GOx and the fast clearance from the body. On the contrary, the PCD/GOx exhibited increasing tumor accumulation within 24 h, where the Cy5.5 signal was much stronger than that of the free GOx-Cy5.5 at the tumor site. It demonstrated the benefits of preparation of nanoclusters in improving the tumor accumulation of enzymes. After monitoring for 24 h, the main tissues were harvested and the Cy5.5 fluorescence was detected (Figure 6B). The GOx-Cy5.5 was mainly distributed in the liver and kidney, showing weak distribution at tumor site. Interestingly, the PCD/GOx-Cy5.5 exhibited strong distribution at the tumor site, significantly higher than that of the free GOx-Cy5.5. The strong tumor site accumulation could be due to the relatively larger size of the nanoclusters, reducing the clearance rate of the enzymes. Therefore, to evaluate the *in vivo* clearance behavior of the free GOx-Cy5.5 and PCD/Cy5.5, mice were injected with the enzyme solutions and the fluorescence intensity in the blood sample was monitored. As shown in Figure 6C, the GOx-Cy5.5 was promptly cleared from the body, whose half-life time (t_1/2_) was only 45 min. Expectantly, the nanoclusters dramatically extended the t_1/2_ of GOx-Cy5.5 to around 4 h. Generally, the size of the nanoparticles was one of the key factors that affected the *in vivo* circulation and biodistribution. Hence, we concluded that the PCD/GOx nanoclusters significantly extended the blood circulation time of GOx and increased the tumor accumulation of the enzymes.

### *In Vivo* PA Imaging

The *in vitro* experiment had demonstrated that ATP was able to activate the catalysis of HRPA or GOx&HRPA and the generation of PA signal. Thus the *in vivo* ATP-lighted tumor PA imaging ability of the nanoclusters was further investigated. The imaging effect of free GOx&HRPA, PCD/HRPA, and the cascade enzyme nanoclusters PCD/GOx&HRPA were compared here. For PA imaging, the background of the mice was first obtained, shown as weak PA signal at tumor site of 0 h (Figure 6D). At 6 h post-injection, the tumor displayed some PA signal of the free GOx&HRPA-treated mice, and it gradually disappeared with the longer observation time at 12 and 24 h, validating the prompt clearance of the free enzymes from the body. Excitingly, the PCD/GOx&HRPA imaging nanoclusters displayed much strong PA signal compared with free GOx&HRPA, and the intensity increased within 24 h, demonstrating that the PCD nanoclusters could significantly improve the PA imaging ability of diagnostic agents. To further evaluate the benefits of the cascade catalysis on amplifying the PA imaging, the GOx-free nanoclusters (PCD/HRPA) were set as control imaging agents. As proved in the result, the much weaker PA signal at tumor site was observed in PCD/HRPA-treated mice versus PCD/GOx&HRPA. The cascade amplifying process could be explained as follows: the PCD nanoclusters improve the tumor delivery of the enzymes and the tumoral ATP could selectively charge the nanoclusters for promoting the release and activation of loaded enzymes, followed by the highly efficient H_2_O_2_ generation by GOx for amplifying the HRP catalysis to activate the ABTS, inducing satisfied PA signal. Therefore, we could conclude that the ATP-charged cascaded enzyme nanoclusters held great potential to be applied for tumor diagnosis.

### *In Vivo* Anti-tumor Efficacy

**Figure 7:**
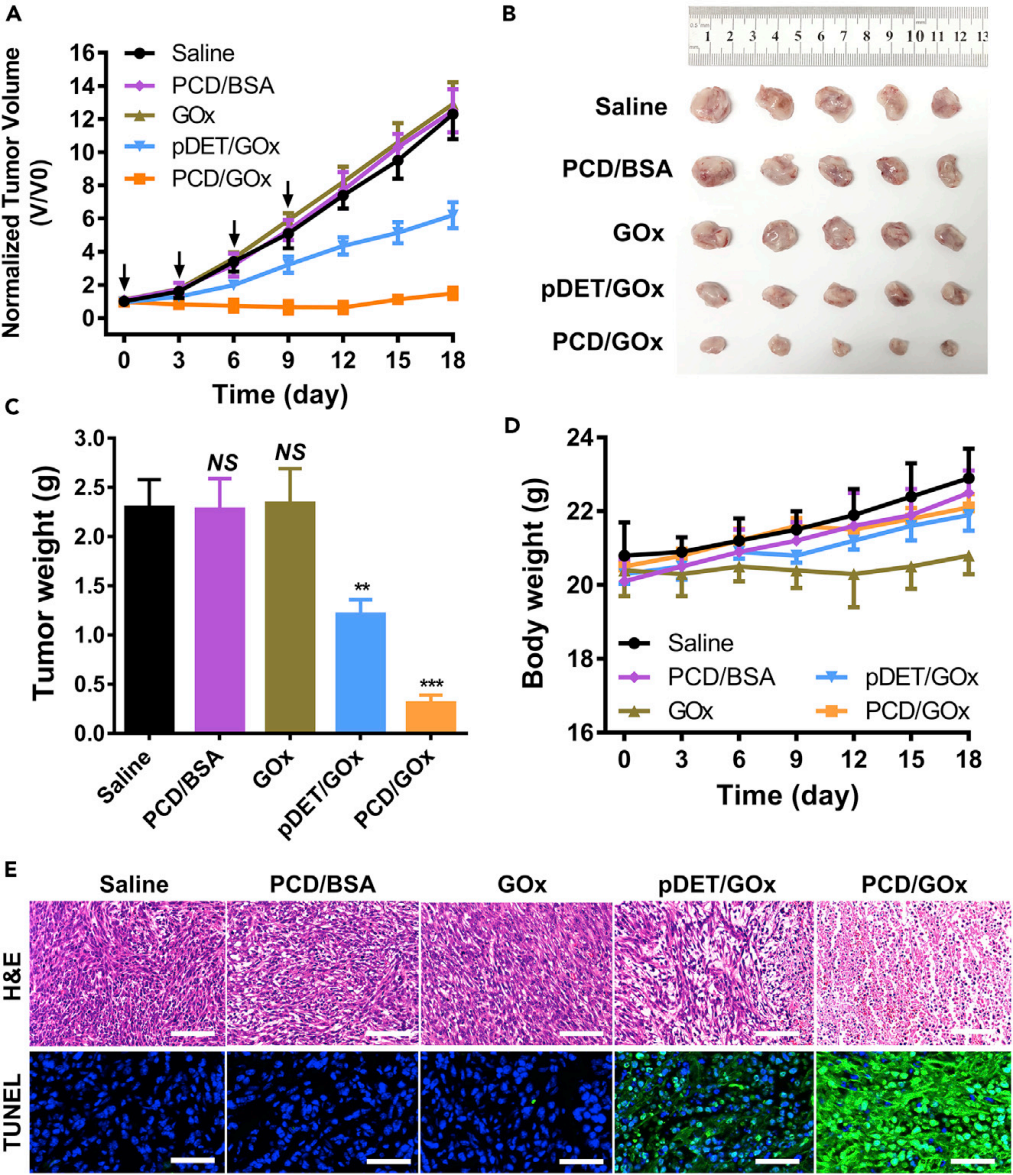
Anti-cancer Efficacy of the PCD/Protein Nanoclusters in 4T1-Bearing Mice (A) Tumor growth curve of 4T1-bearing mice after intravenous injection of saline, PCD/BSA, GOx, pDET/GOx, or PCD/GOx (n = 6, mean ± SD). (B) Photographs of representative ex-tumors imaged at day 18. (C) Tumor weight analysis of the mice harvested at day 18 (n = 6, mean ± SD). NS, not significant different, ∗∗p < 0.01, ∗∗∗p < 0.005 versus Saline group. (D) Body weight monitoring of the mice during treatment (n = 6, mean ± SD). (E) H&E and TUNEL analysis of the tumor sections obtained at day 18. Scale bar (H&E): 200 µm; scale bar (TUNEL): 100 µm. See also Table S1.

The anti-tumor potential of the nanoclusters was also evaluated on subcutaneous 4T1 model by loading cytotoxic proteins. It was reported that many of the enzymes exhibited high activity on tumor cell killing, whereas the *in vivo* application was largely bottlenecked by the inefficient tumor accumulation and uncontrollable catalysis in the blood circulation or the normal tissues. Herein, we desired to evaluate whether the design of ATP-charged nanoclusters could provide breakthrough regarding the dilemma and enable the safe and efficient *in vivo* application of cytotoxic enzymes. In this work, we took GOx as the model enzyme for anti-cancer study by starvation therapy and oxidation therapy. The tumor growth curve (Figure 7A) displayed that the free GOx treatment could not inhibit the tumor, probably because of the weak tumor accumulation and the unspecific catalysis in the circulation. Interestingly, with the pDET encapsulation, the pDET/GOx treatment led to obvious anti-tumor efficacy, demonstrating the necessity of the delivery system. More importantly, after injection of ATP-charged PCD/GOx nanoclusters, the tumor dramatically shrank within 18 days, revealing the advantages of the ATP-charged nanoclusters on *in vivo* enzyme delivery and cancer therapy. Besides, to exclude the potential anti-tumor activity of PCD, PCD/BSA was set as the control, which expectantly did not inhibit the tumor growth, as shown in Figure 7A. At day 18, the representative tumors were harvested and imaged (Figure 7B). The tumor weight was also recorded; the PCD/GOx-treated tumor was only 12.5% versus the saline group and 25.2% of the pDET/GOx-treated mice (Figure 7C). The body weight of the mice was recorded every 3 days. The free GOx-treated mice displayed slight weight loss during the treatment (Figure 7D), which might be caused by the unspecific catalysis of the GOx and the generated systemic toxicity.

To clarify the tumor inhibition mechanism, the tumor tissues were subjected to immunohistochemistry analysis (Figure 7E). H&E staining indicated that the PCD/BSA or GOx could not result in tumor cell apoptosis, whereas necrosis and karyolysis were clear in the sections of PCD/GOx-treated tumor. In addition, pDET/GOx generated cell apoptosis only slightly. Moreover, the TUNEL assay also evidenced the H&E staining analysis; PCD/GOx nanoclusters dramatically initiated the cell apoptosis, observed as strong green fluorescence where the FITC-labeled dUTP was used to stain the fragmented DNA.

To conclude, the PCD/GOx nanoclusters exhibited superior performance in the delivery of cytotoxic enzymes and high activity in anti-cancer therapy.

### Biosafety Evaluation

**Figure 8:**
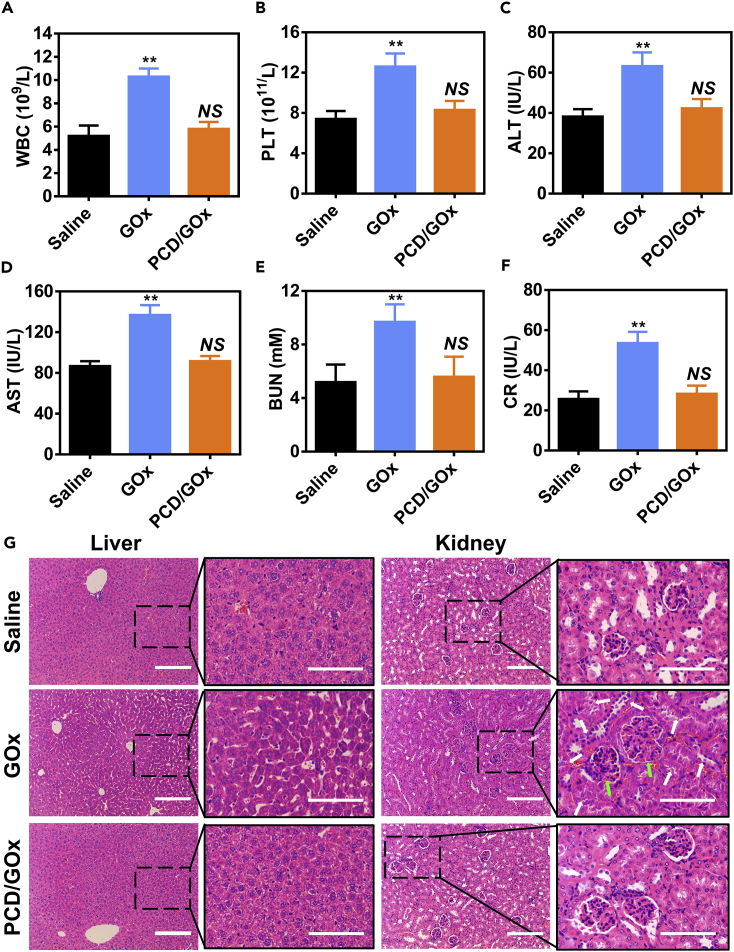
Biosafety Study of PCD/Protein Nanocluster (A–F) Hematological analysis of the mice administered saline, GOx, or PCD/GOx (n = 3, mean ± SD), including (A) the number of white blood cells (WBC), (B) the number of platelets (PLT); and the values of (C) alanine aminotransferase (ALT), (D) aspartate aminotransferase (AST), (E) blood urea nitrogen (BUN), and (F) creatinine (CR). NS, not significant different, ∗∗p < 0.01 versus Saline group. (G) H&E staining of the liver and kidney after treatment with saline, GOx, or PCD/GOx. Green arrows indicate the glomerulus and white arrows indicate the renal tubules. Scale bar: 200 µm. See also Table S1.

The non-selective catalysis of the free enzymes in the blood often resulted in strong systemic toxicity (Ma et al., 2019). In blood circulation, GOx could catalyze the production of toxic H_2_O_2_ due to the presence of the substrate glucose, oxygen, and H_2_O. Here, we conducted the biosafety evaluation of free GOx and PCD/GOx on healthy BALB/c mice after intravenous injection. As shown in Figures 8A and 8B, the white blood cell (WBC) and platelet (PLT) numbers of the GOx-treated mice were significantly higher than those of the saline group, indicating the generation of inflammation and hematotoxicity. On the contrary, the PCD/GOx nanoclusters did not cause the elevation of WBC or PLT, which could be ascribed to the low enzyme activity state in the blood circulation. Apart from hematotoxicity, the GOx was also reported to generate toxicity to the liver and kidney. As shown in Figures 8C and 8D, the free GOx dramatically led to the upregulation of alanine aminotransferase (ALT) and aspartate aminotransferase (AST) in blood sample, which verified the apparent liver toxicity. Furthermore, the elevated creatinine (CR) and blood urea nitrogen (BUN) levels validated that the GOx treatment affected kidney function (Figures 8E and 8F). Expectantly, PCD/GOx nanocluster treatment did not cause the rise of the ALT, AST, CR, or BUN compared with the saline group, proving the biosafety of the nanoclusters. The liver and kidney tissues were also used for immunohistochemical analysis. As depicted in Figure 8G, after GOx treatment, the morphology of the liver cells significantly changed and the nuclei, which were dispersed in the plasma, were not clear, further verifying the liver toxicity of GOx. Besides, the renal interstitial edema indicated by green arrows, disordered renal tubules, and desquamated epithelial cells indicated by white arrows reflected the kidney injury after GOx treatment. In accordance with the blood sample result, PCD/GOx nanoclusters were well biocompatible with the body. The selective catalysis of the nanoclusters maintained the balance between efficient cancer therapy and biosafety as designed in the program.

### Limitations of the Study

PCD with ATP-responsive behavior was successfully applied to modulate the protein activity and enable the intracellular delivery of protein drugs. However, the types of the protein-loading materials should not be ignored. In this study, we only chose the specific linear polypeptide (pDET) as the model material for protein delivery research. The shapes (linear, branched, or cross-linked), dimensions, or molecular weights of the polymers may also have influence on the protein loading and activity modulation. Besides, it is not clear whether this strategy is applicable for inorganic materials (silicon, Fe_3_O_4_, Au, calcium carbonate, etc.), which may need further explorations.

In summary, we demonstrated an ATP-charged nanocluster for controlled intracellular catalysis and cascaded amplification of PA imaging-guided cancer therapy via modulation of the enzyme activity. The PCD nanoclusters shielded the enzyme activity in the blood circulation to reduce the side effects while selectively activating it in the tumoral ATP-abundant environment to obtain highly efficient intracellular catalysis. In addition, the size-switchable strategy among small free enzymes and the relatively larger PCD/enzyme nanoclusters significantly increased the blood circulation time and tumor accumulation. Notably, the preparation of nanoclusters dramatically augmented the PA imaging signal as well as the breast cancer therapeutic effect after loading with HRPA and GOx as the cascaded enzymes. Meanwhile, the PCD/GOx nanoclusters were biocompatible with the mice compared with the toxic free GOx. Thus, the ATP-charged nanoclusters gave an example of bio-responsive enzyme catalysis modulator and could inspire the fabrication of site-specific treatment strategy based on physiological signals. It could also promote the clinical development of protein drugs toward intracellular target by enhancing the therapeutic efficiency and reducing the side effects.

## Methods

All methods can be found in the accompanying Transparent Methods supplemental file.

## Supplemental Information

Document S1. Transparent Methods, Figures S1–S15, and Table S1
