# Supplementary material for: ATP-Charged Nanoclusters Enable Intracellular Protein Delivery and Activity Modulation for Cancer Theranostics
Source: iScience. 2020 Jan 31;23(2):100872. doi: 10.1016/j.isci.2020.100872 (PMC7016238; doi:10.1016/j.isci.2020.100872)
Supplement: Document S1. Transparent Methods, Figures S1–S15, and Table S1 [file mmc1.pdf]

iScience, Volume *23*

Supplemental Information

ATP-Charged Nanoclusters Enable Intracellular Protein Delivery and Activity Modulation for Cancer Theranostics

Zhanwei Zhou, Qingyan Zhang, Ruoxi Yang, Hui Wu, Minghua Zhang, Chenggen Qian, Xiangzhong Chen, and Minjie Sun

Transparent Methods

Materials

mPEG-NH_2 (MW: 2000 Da) was purchased from Peng Sheng Biological Company (Shanghai, China). β-benzyl-L-aspartate-*N*-Carboxy-anhydride (BLA-NAC) was obtained from Bide Pharmatech Ltd (Shanghai, China). 3-Acrylamidophenylboronic Acid (APBA), 4-carboxyphenylboronic acid (PBA), 4-Carboxy-3-fluorophenylboronic acid (FPBA), EDC. HCl, NHS, ABTS, HRP and all of the dry solvent were purchased from Aladdin chemical regent company (Shanghai, China). GOx (40 unit/mg) was purchased from TCI company (Shanghai, China) and β-galactosidase (β-Gal, 205 unit/mg) was from Beijing Solarbio Science & Technology Co., Ltd. MTT, TUNEL assay kit and ROS detection kit were purchased from KeyGEN BioTECH (Nanjing, China). RNase A (60 KU/mg) was purchased from Shanghai Macklin Biochemical Co., Ltd.

Synthesis of mPEG-PBLA

The synthesis of mPEG-PBLA was based a ring opening polymerization of BLA-NAC (Lee et al., 2008). mPEG-NH_2 as the macro-initiator (designed degree of polymerization (DP) = 100). The reaction was performed in a mixture solution of DMF/DCM at 1:10 v/v ratio. Briefly, mPEG-NH_2 (MW: 2000, 0.0521 mmol) was dissolved in 6 mL mixture solvent. The BLA-NCA (5.75 mmol) was dispersed in 10 mL mixture solvent and subsequently added into the PEG-NH_2 solution. The polymerization reaction was stirred at 35 ℃ for 48 h under the N_2 atmosphere. After reaction, the resulting product was precipitated into diethyl ether (200 mL). The crude

precipitate was washed with diethyl ether (10 mL × 2) to obtain the final product as a white powder. The chemical structure of PEG-PBLA was characterized by ^{1}H-NMR (Solvent: d6-DMSO).

Synthesis of mPEG-PDET (pDET)

Briefly, the PEG-PBLA (400 mg) was dispersed in NMP (10 mL). Subsequently, diethylenetriamine (DET, 50 equivalents to BLA unit) was added to the PEG-PBLA solution and the reaction was stirred at 0 °C for 1 h. After reaction, the resulting solution was added dropwise into aqueous acetic acid solution (10%, 10 mL) to obtain neutralized solution. The excess DET was removed by dialysis against DW for 24 h and the final product of white powder was obtained after lyophilization. The chemical structures were validated by ^{1}H-NMR (Solvent: D_2O).

Synthesis of PAD

The synthesis of PAD was based on the Michael addition reaction. Briefly, calculated 3-acrylamide phenylboronic acid (19 mg, 38mg and 72 mg) was dissolved in a mixture solution (methanol: water = 6: 4) and added to the pDET (210 mg) solution dissolved in the mixture solution. The reaction was stirred at 50 °C for 48 h under N_2 protection. Finally, the obtained reaction mixture was dialysis against DW for 24 h. The chemical structures of PAD were validated by ^{1}H-NMR (Solvent: D_2O). The substitution degree (DS) of the 3-acrylamide phenylboronic acid to pDET was calculated to be 8.3%, 16.9% and 27.6% by NMR spectrum, which was named as PAD1, PAD2 and PAD3 separately.

Synthesis of PCD

The PCD synthesis was based on amidation reaction. 4-carboxyphenylboronic acid (PBA, 24 mg, 48 mg and 96 mg) was dissolved in DW and the pH was adjusted to 5.5 by adding 1 M NaOH. Subsequently, the carboxy of PBA was activated by EDC and NHS for 1 h under vigorous stirring. Then, the PBA solution was dropwise added into the pDET (210 mg) solution and the pH was adjusted to 5.5 - 6 by 1 M HCl. It was reacted at RT for 24 h. Finally, the product was obtained by dialysis and lyophilization. The chemical structures of PCD was characterized by ^{1}H-NMR (Solvent: D_2O). The DS of PBA to pDET was calculated to be 7.8%, 14.6% and 29.3% by NMR spectrum, named as PCD1, PCD2 and PCD3 separately.

Synthesis of FPCD

The synthesis procedure of FPCD was similar as PCD where 3-carboxy-4-fluorophenylboronic acid (FPBA) was used to replace the 4-carboxyphenylboronic acid. Here, calculated FPBA (27.6 mg, 55.2 mg and 110.4 mg) was reacted with pDET for 24 h in pH 5.5 water solution. The obtained product was analysed by ^{1}H-NMR (Solvent: D_2O). The DS of PBA to pDET was calculated to be 7.3%, 15.8% and 26.7% by NMR spectrum, named as FPCD1, FPCD2 and FPCD3 separately.

Preparation and characterization of nanoclusters

The nanoclusters were prepared by simple mixing of the polycations with proteins at calculated weight ratio. Here, bovine serum albumin (BSA) was firstly taken as the model protein for evaluating the formulations with different polycations modified with series degree of PBA (pDET, PAD1, PAD2, PAD3, PCD1, PCD2, PCD3, FPCD1, FPCD2 and FPCD3).

The particle size and zeta potential of BSA and the above nanoclusters were measured by Malvern Mastersizer.

The morphology of pDET/BSA, PAD/BSA, PCD/BSA and FPCD/BSA was performed on H-600 transmission electron microscope (TEM) (Hitachi, Japan). The polycations with high PBA modification ratio were applied here for TEM observation.

Synthesis of FITC labelled proteins and fluorescence quenching assay

For BSA-FITC synthesis, 200 mg BSA was dissolved in Na_2CO_3/$NaHCO_3$ buffer (pH = 9). Subsequently, 20 mg fliorescein isothiocyanate (FITC, dissolved in 200 μL DMSO) was dropwise added in the BSA solution. The reaction was performed for 12 h in the dark environment, followed by dialysis against DW for 12 h (1 L × 3) and lyophilization. The RNase A-FITC and GOx-FITC were synthesised with the similar method as BSA.

The fluorescence spectrums of BSA-FITC with or without PCD involvement were obtained by fluorescence spectrophotometer (ex: 480 nm, em: 500-700 nm). The concentration of BSA-FITC was 20 μg/mL for each sample. The PCD was pre-mixing with BSA-FITC at various weight ratios (pDET : BSA) of 0.25, 0.5, 0.75, 1 and 2.

Circular Dichroism (CD) measurements

The secondary structure of the proteins and nanoclusters (BSA and PCD/BSA were taken as example) was measured by a circular dichroism (CD) spectrometer J-815 (JASCO, Japan).

Binding mechanism analysis by fluorescence recovery

The assembly/disassembly of nanoclusters were evaluated by fluorescence quenching and fluorescence recovery. The fluorescence intensity of BSA-FITC, PCD/BSA-FITC and PCD/BSA-FITC with Triton X-100 (0.1%), Tween 20 (0.1%) or heparin (2%) was detected by fluorescence spectrophotometer (ex: 480 nm, em: 520 nm).

Stability study of PCD/BSA nanoclusters

For stability study, the PCD/BSA nanoclusters were incubated with DW, rat blood serum, DMEM cell culture medium or pH 6.8 PBS buffer, followed by monitoring the particle size by Malvern Mastersizer within 24 h.

ATP-triggered charge reversal and BSA release study

The zeta potential of pDET/BSA, PAD/BSA, PCD/BSA and FPCD/BSA was monitored by Malvern Mastersizer after incubation with 0-4 mM ATP for 5 min separately where the unreacted ATP was removed by untrafitration.

The release of BSA-FITC was evaluated by the fluorescence recovery of the nanoclusters, detected at ex 488 nm, em 520 nm. All of the candidate nanoclusters were treated with 0.4 mM – 4 mM ATP and the fluorescence intensity were measured. The released ratio was calculated according to the reported method[28]. We assumed that the total fluorescence intensity (I) were the sum of the intensity of the free BSA-FITC (I_f) and that of the intensity of the BSA-FITC inside the nanoclusters (I_m), the the following equation:

$$I = I_f + I_m = I_{f0} \times \frac{x}{100} + I_{m0} \times \frac{100-x}{100}$$ Formula (1)

(I_{f0}: the fluorescence intensity of free BSA-FITC without any polymer complexation;

I_{m0}: the initial fluorescence intensity of BSA-FITC in the nanoclusters

x: the percentage of released BSA-FITC)

Besides, the responsiveness of PCD/BSA-FITC nanoclusters to the ATP analogues was also evaluated, such as ADP (adenosine diphosphate), AMP (adenosine monophosphate), adenosine, dATP (deoxyadenosine triphosphate), GTP (guanosine triphosphate), CTP (cytidine triphosphate) and UTP (uridine triphosphate). The PCD/BSA-FITC was incubated with 4 mM analogues for 5 min, and then the fluorescence intensity was detected. The release ratio was calculated as mentioned above.

ATP charged enzyme activation and high efficiently catalysis

The ATP modulated enzyme activity of PCD/Enzymes nanoclusters was evaluated on 4 model enzymes with different molecular weight (MW) and isoelectric point (PI): β-Gal (MW: 430 kDa, PI: 5), RNase A (MW: 13.7 kDa, PI: 9.6), GOx (MW: 80 kDa, PI:4.6) and HRP (MW: 40 kDa, PI: 7.2).

For β-Gal activity measuring, the substrate solutions of *X-Gal* were incubated with β-Gal, pDET/β-Gal or PCD/β-Gal with 0 - 4 mM ATP for 1 h, where the free β-Gal was set as positive control with 100% enzyme activity. Then, the resulting blue product was centrifuged and dissolved with DMSO, followed by detecting the absorbance at 633 nm by microplate reader. The relative enzyme activity (REA) of β-Gal was calculated by comparing with free β-Gal.

For RNase A activity measuring, siRNA was incubated with RNase A, pDET/RNase A or PCD/RNase A with 0-4 mM ATP for 1 h. Subsequently, the

remained siRNA was analysed by agarose gel electrophoresis. The REA of RNase A was calculated according to the formula (2) which was referred to the siRNA degradation degree.

$REA_{(RNase\ A)} = (siRNA_{total} - siRNA_{remained}) / siRNA_{total} \times 100\%$ Formula (2)

where the $siRNA_{total}$ means the total amount of siRNA, $siRNA_{remained}$ means the undegraded siRNA.

For GOx activity measuring, the glucose solution (100 mg/mL) was incubated with GOx, pDET/GOx or PCD/GOx with 0 - 4 mM ATP for 4 h. The catalysed H_2O_2 was detected by the H_2O_2 kit (KeyGEN, Nanjing). The REA of GOx was calculated by comparing the H_2O_2 production amount with free GOx.

The HRP@ABTS (HRPA) was synthesised by simple mixing of HRP (50 mg) and ABTS (50 mg) and stirring at RT for 24 h. The unencapsulated ABTS was removed by ultrafiltration. For HRP activity measuring, the substrate H_2O_2 solution was incubated with HRPA, pDET/HRPA or PCD/HRPA with 0-4 mM ATP for 1 h. Afterward, the UV-Vis spectrum and the absorbance at 730 nm of the resulting solutions were detected by Ultraviolet-Visible Spectrophotometer.

Cell uptake and tumor cell killing.

The cell uptake of free BSA-FITC and all of the nanoclusters was evaluated by flowcytometry (FCM) and confocal laser scanning microscopy (CLSM). Briefly, for FCM analysis, the 4T1 cells were seeded into 24 well plate. After growing for 24 h, the free BSA-FITC (10 μg/mL) and the BSA-FITC loaded nanoclusters were incubated with cells for 4 h. The cells were then washed with PBS twice, followed by collected

by trypsin treatment and FCM analysis. For CLSM observation, the cells were seeded in confocal dishes. Free BSA-FITC (10 μg/mL) and the representative nanoclusters (pDET/BSA-FITC, PCD2/BSA-FITC and PCD3/SA-FITC) were incubated with 4T1 cells, L02 cells or MCF-7 cells followed by washed twice with PBS, fixed by 4% polyformaldehyde and stained with DAPI. The observation of the uptake of RNase A-FITC (5 μg/mL) and GOx-FITC (5 μg/mL) was performed as the same protocol as BSA-FITC.

The cell viability of 4T1 cells and L02 cells were evaluated by MTT assay. 4T1 cells and L02 cells were seeded into 96 well plates and incubated for 24 h in the CO_2 incubator. Series concentration of pDET, PCD, free enzyme, pDET/enzyme and PCD/enzyme (RNase A or GOx) were incubated with cells for 4 h in the incomplete medium. Then, the enzyme contained medium was replaced with complete DMEM medium (10% FBS) for another 20 h. Finally, 20 μL MTT (5 mg/mL) was added to each well. The MTT contained medium was removed after incubation in the incubator for 4 h and the frozen was dissolved using DMSO. The absorbance (492 nm) of each well was detected by microplate reader.

Intracellular ATP dependent protein release and enzyme activation

The FRET (Fluorescence Resonance Energy Transfer) technology was applied to evaluate the ATP triggered disassembly of nanoclusters and release of proteins (Zhou et al, 2019). BSA was labelled with FITC and acted as the donor of the FRET pair. PCD was labelled with RhB and acted as the receptor. The excitation wavelength of the detection was 488 nm and emission wavelength were among 500 nm to 700 nm. The

fluorescence spectrum was detected with the increasing ATP added into PCD/BSA-FITC solution.

The intracellular ATP dependent protein release was also evaluated by FRET. Here, iodoacetic acid (IAA, 100 μM) was used to deplete the intracellular ATP. Briefly, the 4T1 cells were incubated with PCD/BSA-FITC for 2 h. Afterward, the proteins contained medium was replaced with fresh medium or IAA contained medium and incubated for another 6 h, followed by CLSM observation (Zeiss 700, German). The observation excitation wavelength for FITC and RhB was 488 nm and the emission range for FITC was 500-550 nm and for RhB was 550-650 nm.

The activity of intracellular HRP was evaluated by using a fluorescence substrate Amplex Red. The non-fluorescent Amplex Red could be catalysed to fluorescent resorufin (ex: 530-560 nm, em: 590 nm) in the presence of H_2O_2. The 4T1 cells were seeded in the confocal dishes and treated with the similar procedure as FRET experiment. Free HRP or PCD/HRP were incubated with cells for 2 h. Then, the PCD/HRP contained medium was replaced with fresh medium, IAA contained medium or NaN_3 contained medium. After incubation for 6 h, the cells were washed with PBS for 3 times and incubated with Amplex Red (50 μM) and H_2O_2 (500 μM) contained PBS solution for 30 min in the incubator. The stained cells were washed and observed by CLSM (ex: 555 nm, em: 560-650 nm).

The intracellular activity of β-Gal was evaluated by in situ X-Gal staining kit. The β-Gal could catalyse the substrate X-Gal from colourless to blue. The 4T1 cells were seeded in 6 well plates and grew for 24 h. The cells were then treated with free β-Gal

or PCD/β-Gal as the similar procedure of HRP. Finally, the cells were stained by X-Gal according to the standard protocols of the kit (stained for 2 h at 37 °C in the CO_2 free incubator), washed 3 times by PBS and imaged by microscopy. The relative enzyme activity was also quantitatively analysed by image J.

The PCD/GOx nanoclusters were transfected under complex conditions to evaluate the stability during transfection. The different conditions contained temperature (25 °C, 37 °C, 43 °C), pH (7.4, 6.5, 5.5) or serum (0%, 10%, 30%). Briefly, the PCD/GOx was incubated under certain conditions for 4 h, flowed by staining with DCFH-DA. After 30 min, the DCFH-DA was washed and the images of green fluorescence were obtained by fluorescence microscopy.

Animals

Female BALB/c mice (18 - 22 g) were bought from Yangzhou University. All the animal experiments were performed in compliance with the Guide for Care and Use of Laboratory Animals and were approved by China Pharmaceutical University.

***In vivo* biodistribution, tumor accumulation and blood circulation of PCD/GOx-Cy5.5**

The *in vivo* biodistribution, tumor targeting and blood circulation of the free GOx and PCD/GOx nanoclusters were studied by labelling the GOx with Cy5.5 to track the enzymes. The 4T1 bearing mice were randomly divided into 2 groups (n=3): GOx-Cy5.5 and PCD/GOx-Cy5.5. When the tumor size reached 200 mm^3, the mice were administrated by *i.v.* injection with Cy5.5 dose of 0.5 mg/kg. The tumor accumulation was observed by Caliper IVIS Lumina II *in vivo* image system at 6 h, 12 h and 24 h.

After observation, the mice were sacrificed and the main tissues (heart, liver, spleen, lung, kidney and tumor) were harvested. The fluorescence intensity of Cy5.5 in the tissue lysates was detected by fluorescence spectrophotometer. For blood circulation monitoring, the mice were given the same treatment as mentioned above and the blood samples were collected at time intervals (0 h, 0.25 h, 0.5 h, 1 h, 2 h, 4 h, 8 h, 12 h, 24 h and 48 h). Fluorescence intensity of Cy5.5 in the blood sample was detected and calculated according to the standard curve.

***In vivo* PA imaging**

The 4T1 tumor bearing mice were randomly divided into 3 groups (n=3) and scanned by Vevo LAZR Imaging System pre-injection to obtain the background signal of the mice. After that, the mice were administrated by *i.v.* injection of GOx & HRPA, PCD/HRPA and PCD/GOx & HRPA (200 μL, 6 mg/mL HRP and 0.3 mg/mL GOx) at exciting wavelength of 800 nm.

***In vivo* anti-tumor efficacy**

The 4T1 xenograft tumor model was constructed on BALB/c mice (1×10^6 per mouse). When the tumor size reached 150 mm^3, mice were randomly divided into 5 groups: 1) Saline, 2) PCD/BSA, 3) GOx, 4) PDET/GOx, 5) PCD/GOx (GOx dose: 0.5 mg/kg). The mice were administrated the formulations by *i.v.* injection at Day 0, 3, 6 and 9. Tumor size and body weight were recorded every 3 days. The tumor volume was calculated as the following equation: Tumor volume (mm^3) = $0.5 \times length \times width^2$. At day 18, the mice were sacrificed and the tumors were harvested from the body. The representative ex-tumors were imaged and the tumor weight was recorded. All of the

tumor samples were then embedded in paraffin, sliced and stained with H&E. For tumor apoptosis analysis, the nucleic of tumor cells were stained with DAPI and apoptosis cells were stained with dUTP-FITC for TUNEL analysis.

Biosafety evaluation study

For biosafety study, the healthy BALB/c mice were randomly divided into 3 groups (n = 3). The mice were administrated with saline, GOx or PCD/GOx (GOx dose: 0.5 mg/kg) every three days for 4 treatments. At day 12, the mice were sacrificed, the blood samples and main organs were taken for routine blood test and H&E staining.

Statistical analysis

All statistical analyses were performed using GraphPad Prism version 6 software. Data from the experiments were performed for three times or over three times. The results were expressed as the mean value ± standard deviation (Mean ± SD). A two-tailed student's t-test was performed for statistical analysis of the difference between the two groups. $P\ value < 0.05$ was considered statistically significant between the data sets, where all significant values were indicated as follows: $*p< 0.05$, $**p< 0.01$, $***p< 0.001$.

Supplemental References

Lee, Y., Miyata, K., Oba, M., Ishii, T., Fukushima, S., Han, M., Koyama, H., Nishiyama, N., and Kataoka, K. (2008). Charge‐Conversion Ternary Polyplex with Endosome Disruption Moiety: A Technique for Efficient and Safe Gene Delivery. Angew. Chem. Int. Ed. *47*, 5163-5166.

Zhou, Z., Liu, Y., Zhang, M., Li, C., Yang, R., Li, J., Qian, C., and Sun, M. (2019). Size Switchable Nanoclusters Fueled by Extracellular ATP for Promoting Deep Penetration and MRI‐Guided Tumor Photothermal Therapy. Adv. Funct. Mater. 1904144.

Supplementary Figures and Tables

Table S1. Related to **Figure 1-8**. Abbreviations and the corresponding full names in the manuscript.

**Abbreviations**	**Full names**
ATP	adenosine triphosphate
ABTS	2, 2'-azino-bis(3-ethylbenzothiazoline-6-sulfonic acid
ADP	adenosine diphosphate
ALT	alanine aminotransferase
AMP	adenosine monophosphate
AST	aspartate aminotransferase
BSA	bovine serum albumin
BUN	urea nitrogen
CD	circular dichroism
CLSM	confocal laser scanning microscope
CR	creatinine
CTP	cytidine triphosphate
dATP	deoxyadenosine triphosphate
DS	degree of substitution
DW	deionized water
FCM	flow cytometry
FITC	fluorescein isothiocyanate
FPCD	3-carboxy-4-fluorophenylboronic acid modified pDET
FRET	fluorescence resonance energy transfer
GOx	glucose oxidase
GTP	guanosine triphosphate
H_2O_2	hydrogen peroxide
HRP	horseradish peroxidase
IAA	Iodoacetic acid
MFI	mean fluorescence intensity
MW	molecular weight
NIRF	near infrared fluorescence
PA	photoacoustic
PAD	3-(Acrylamido) phenylboronic acid modified Pdet
PBA	phenylboronic acid
PCD	4-Carboxyphenylboronic acid modified pDET
pDET	mPEG-b-poly(2-[(2-aminoethyl)amino]ethylaspatamide
PI	isoelectric point
PLT	platelets
RhB	rhodamine B
RNase A	ribonuclease A

STR	Shield-Transport-Recover
UTP	uridine triphosphate
WBC	white blood cells
β-Gal	β-Galactosidase

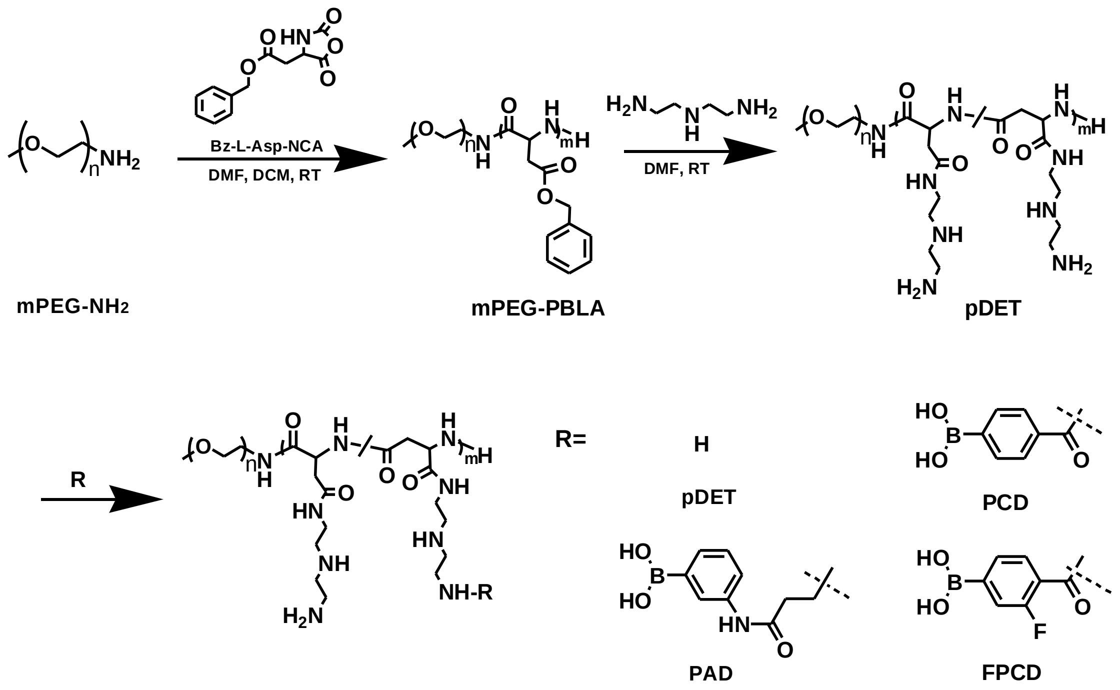

Figure S1. Related to **Figure 1**. The synthesis procedure of mPEG-PBLA, pDET, PAD, PCD and FPCD.

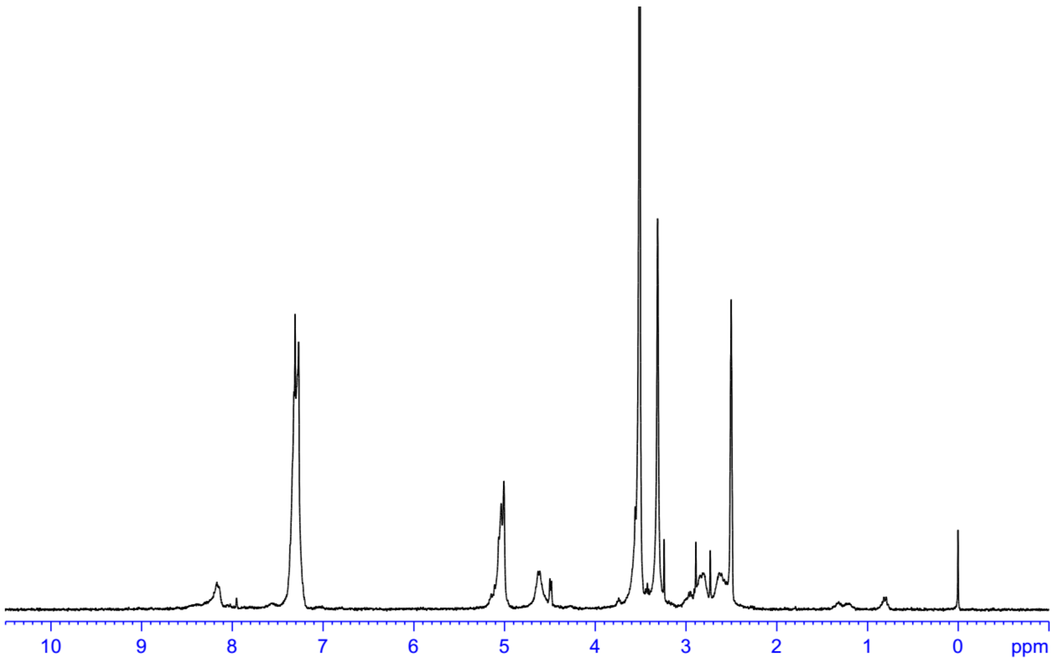

Figure S2. Related to **Figure 1**. ^{1}H-NMR spectrum of mPEG-PBLA in d6-DMSO.

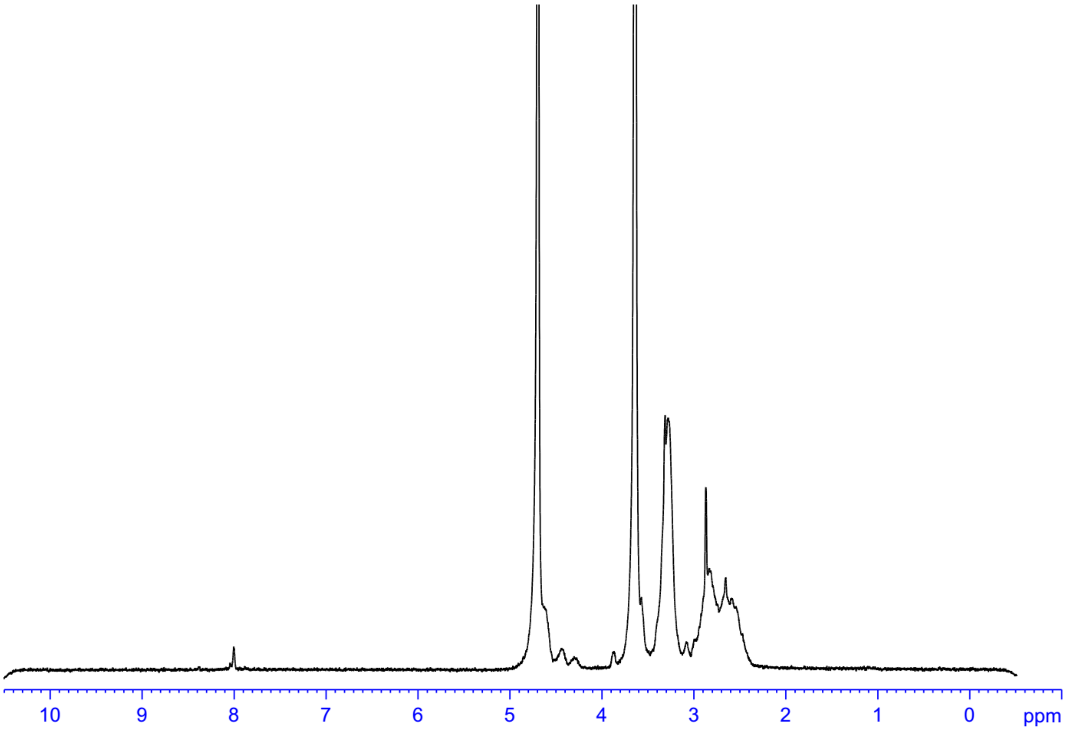

Figure S3. Related to **Figure 1**. ^{1}H-NMR spectrum of pDET in D_2O.

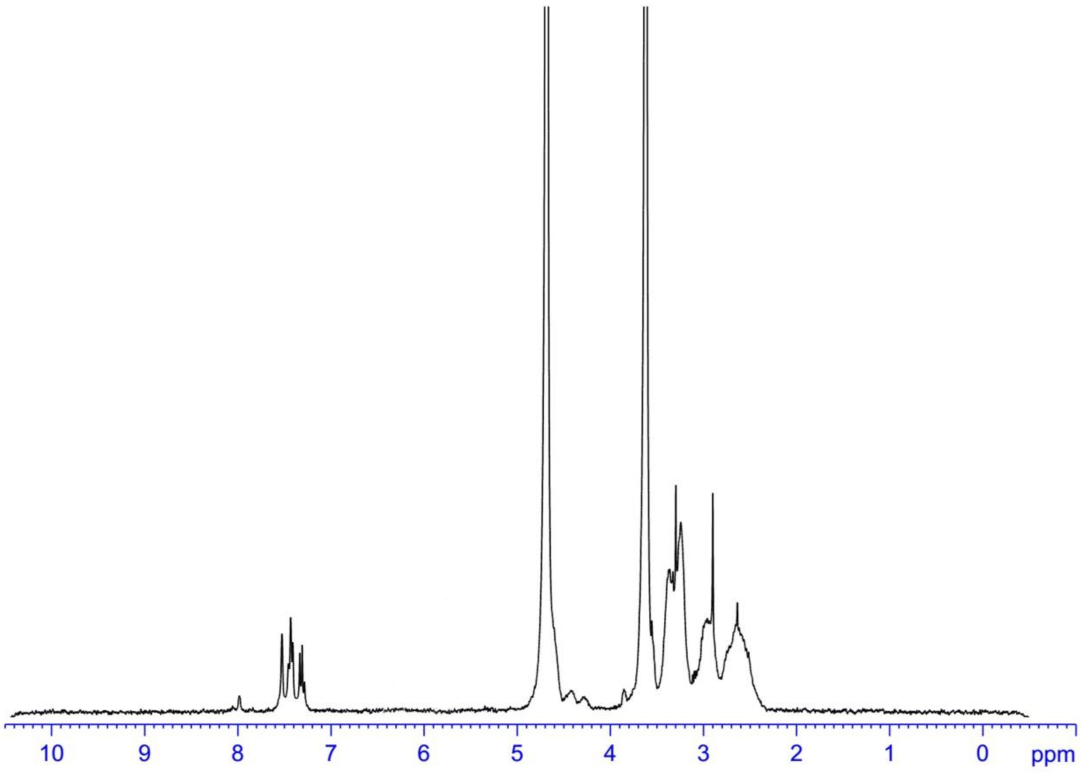

Figure S4. Related to **Figure 1**. ^{1}H-NMR spectrum of PAD in D_2O.

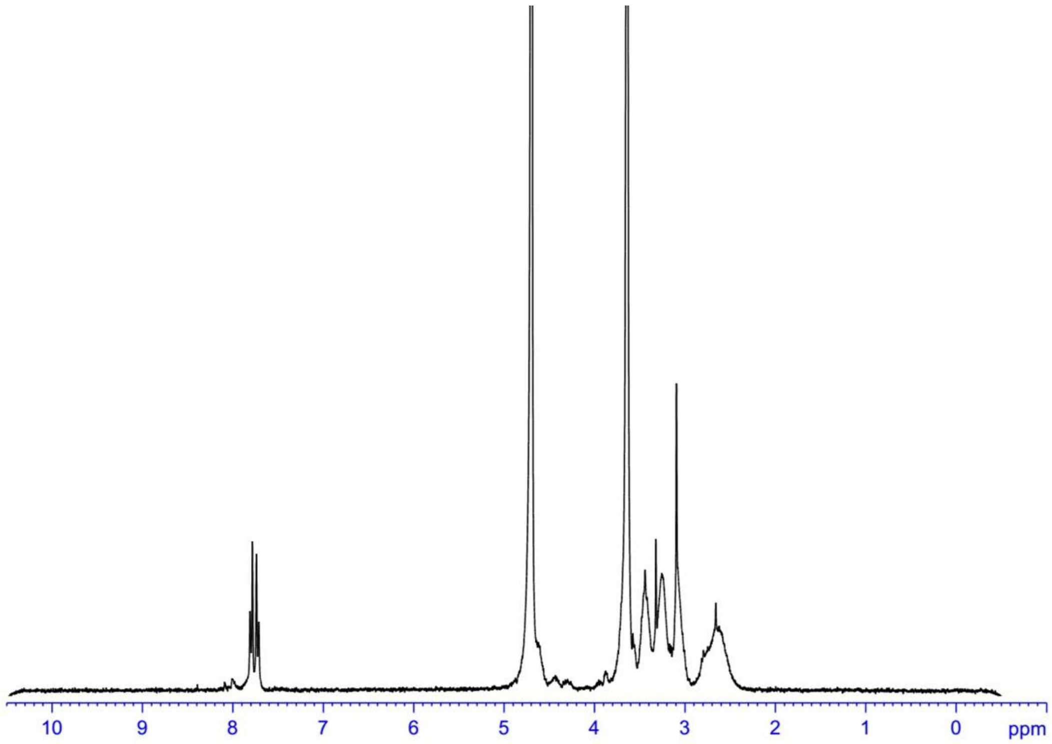

Figure S5. Related to **Figure 1**. ^{1}H-NMR spectrum of PCD in D_2O.

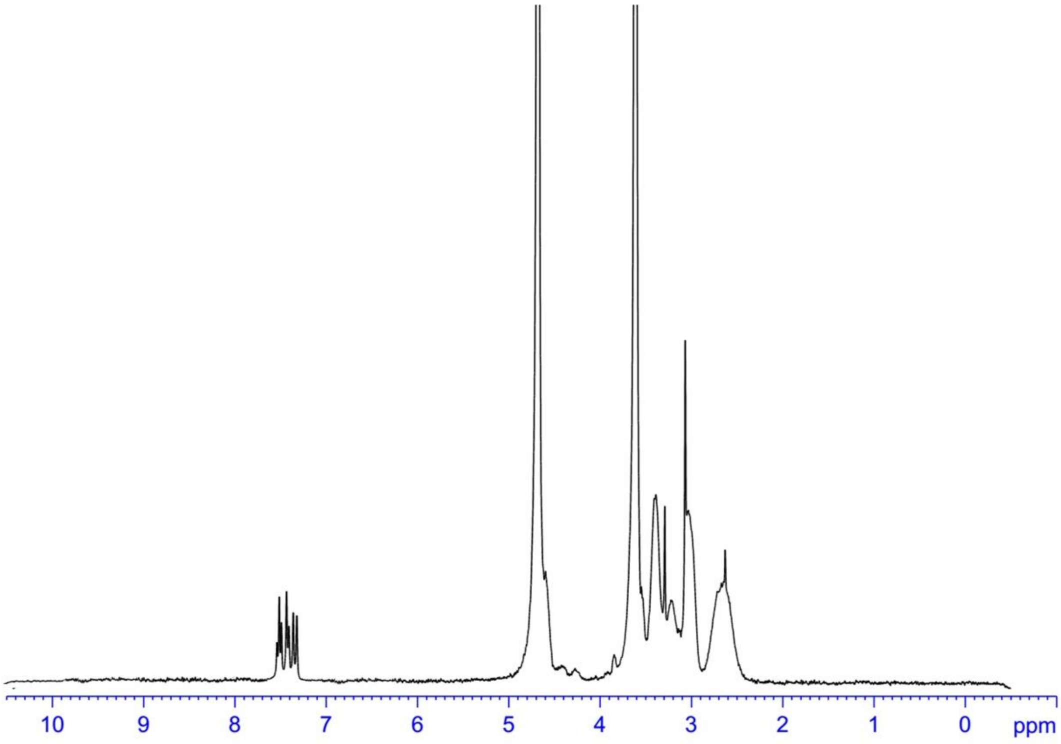

Figure S6. Related to **Figure 1**. ^{1}H-NMR spectrum of PFCD in D_2O.

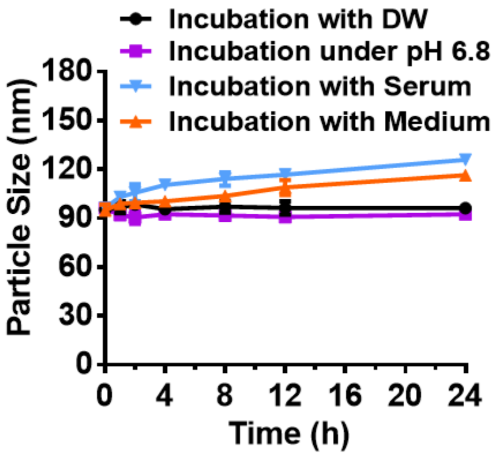

Figure S7. Related to **Figure 1**. Stability of the PCD/BSA nanoclusters incubated with DW, serum, medium or pH 6.8 environment (n=3, Mean ± SD).

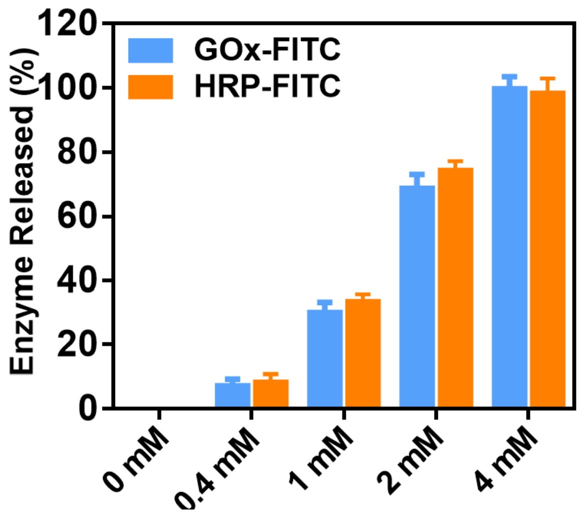

Figure S8. Related to **Figure 2**. Release study of PCD/GOx&HRP nanocluster by separately detecting the fluorescence recovery of FITC labelled enzymes (PCD/GOx-FITC&HRP and PCD/GOx&HRP-FITC) (n=3, Mean ± SD).

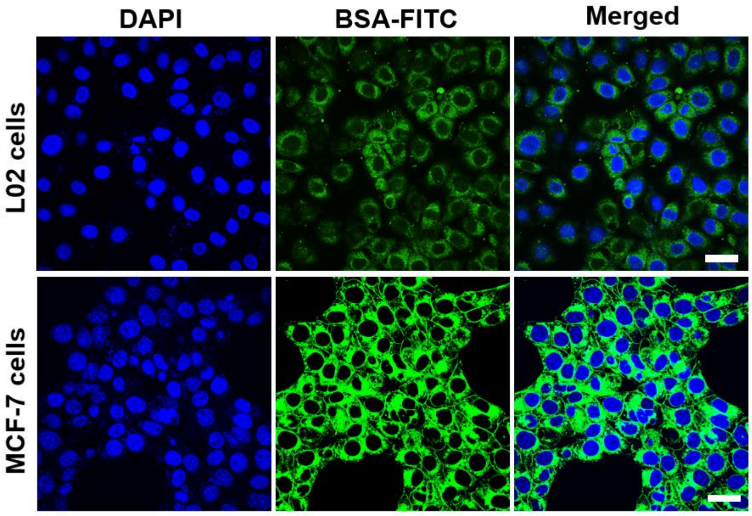

Figure S9. Related to **Figure 4**. Cell uptake of PCD/BSA-FITC nanoclusters on L02 cells and MCF-7 cells. Scale bar: 20 μm.

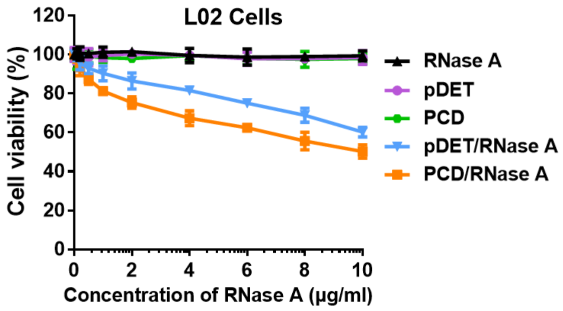

Figure S10. Related to **Figure 4**. Cell viability of L02 cells incubated with pDET, PCD, RNase A, pDET/RNase A and PCD/RNase A (n=3, Mean ± SD).

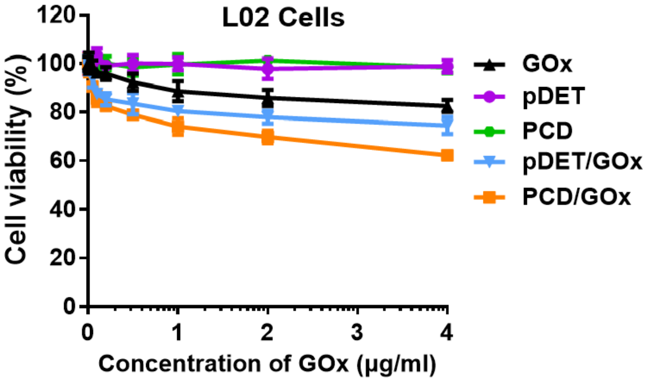

Figure S11. Related to **Figure 4**. Cell viability of L02 cells incubated with pDET, PCD, GOx, pDET/GOx and PCD/GOx (n=3, Mean ± SD).

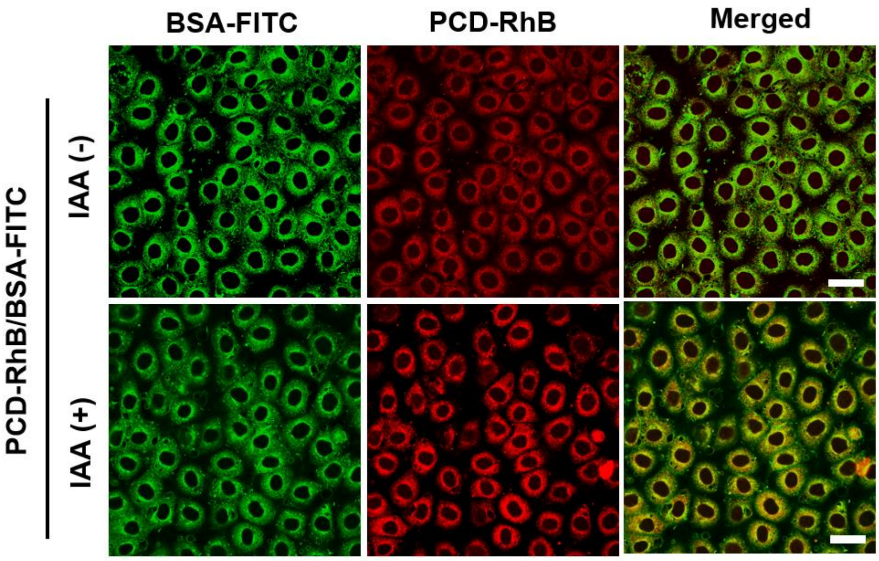

Figure S12. Related to **Figure 5**. Intracellular protein release by FRET technology on L02 cell lines, where the FITC acted as the donor and the RhB acted as the acceptor (IAA: ATP generation inhibitor). Scale bar: 20 μm.

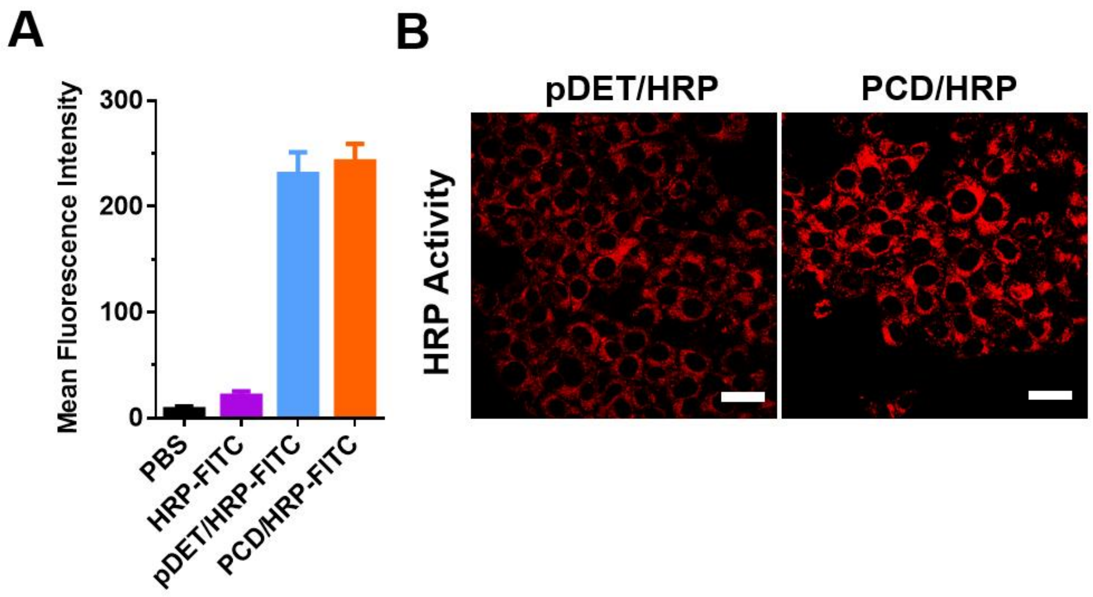

Figure S13. Related to **Figure 5**. (A) Cell uptake study of HRP-FITC (50 μg/ml), pDET/HRP-FITC (50 μg/ml) and PCD/HRP-FITC (10 μg/ml). (B) CLSM images of 4T1 cells transfected with pDET/HRP or PCD/HRP and processed with H_2O_2 and Amplex Red. Scale bar: 20 μm.

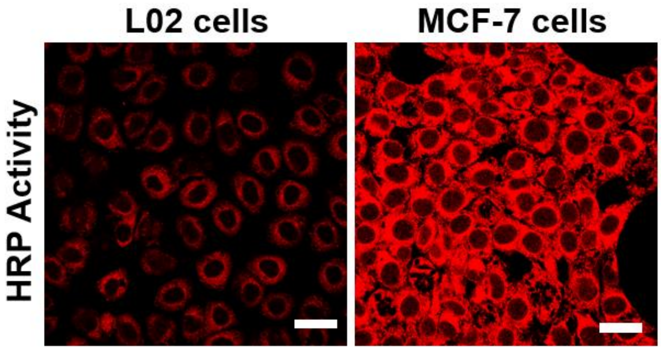

Figure S14. Related to **Figure 5**. CLSM images of L02 cells or MCF-7 cells transfected with PCD/HRP and processed with H_2O_2 and Amplex Red. Scale bar: 20 μm.

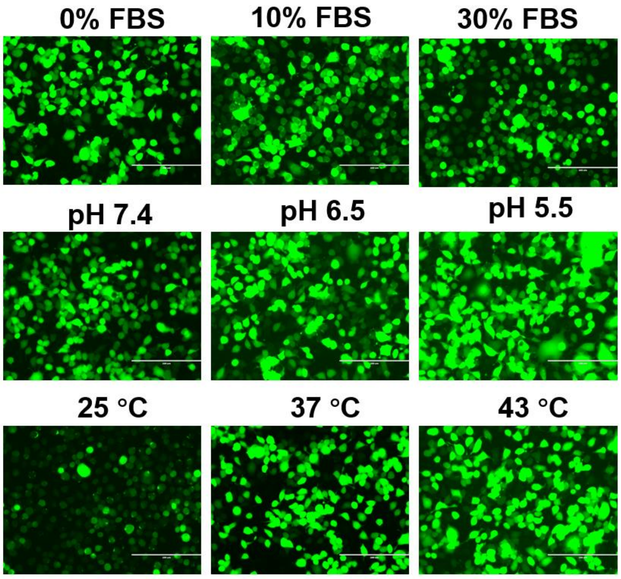

Figure S15. Related to **Figure 5**. Intracellular ROS level of 4T1 cells after transfected with PCD/GOx under various conditions: temperature (25 °C, 37 °C, 43 °C), pH (7.4, 6.5, 5.5) or serum (0%, 10%, 30%). Scale bar: 200 μm.
